# Supplementary material for: Within-Subtype HIV-1 Polymorphisms and Their Impacts on Intact Proviral DNA Assay (IPDA) for Viral Reservoir Quantification
Source: Viruses. 2025 Oct 31;17(11):1453. doi: 10.3390/v17111453 (PMC12656820; doi:10.3390/v17111453)
Supplement: Supplementary file 1 [file viruses-17-01453-s001.zip › viruses-3942942-supplementary.pptx]

## Slide 1
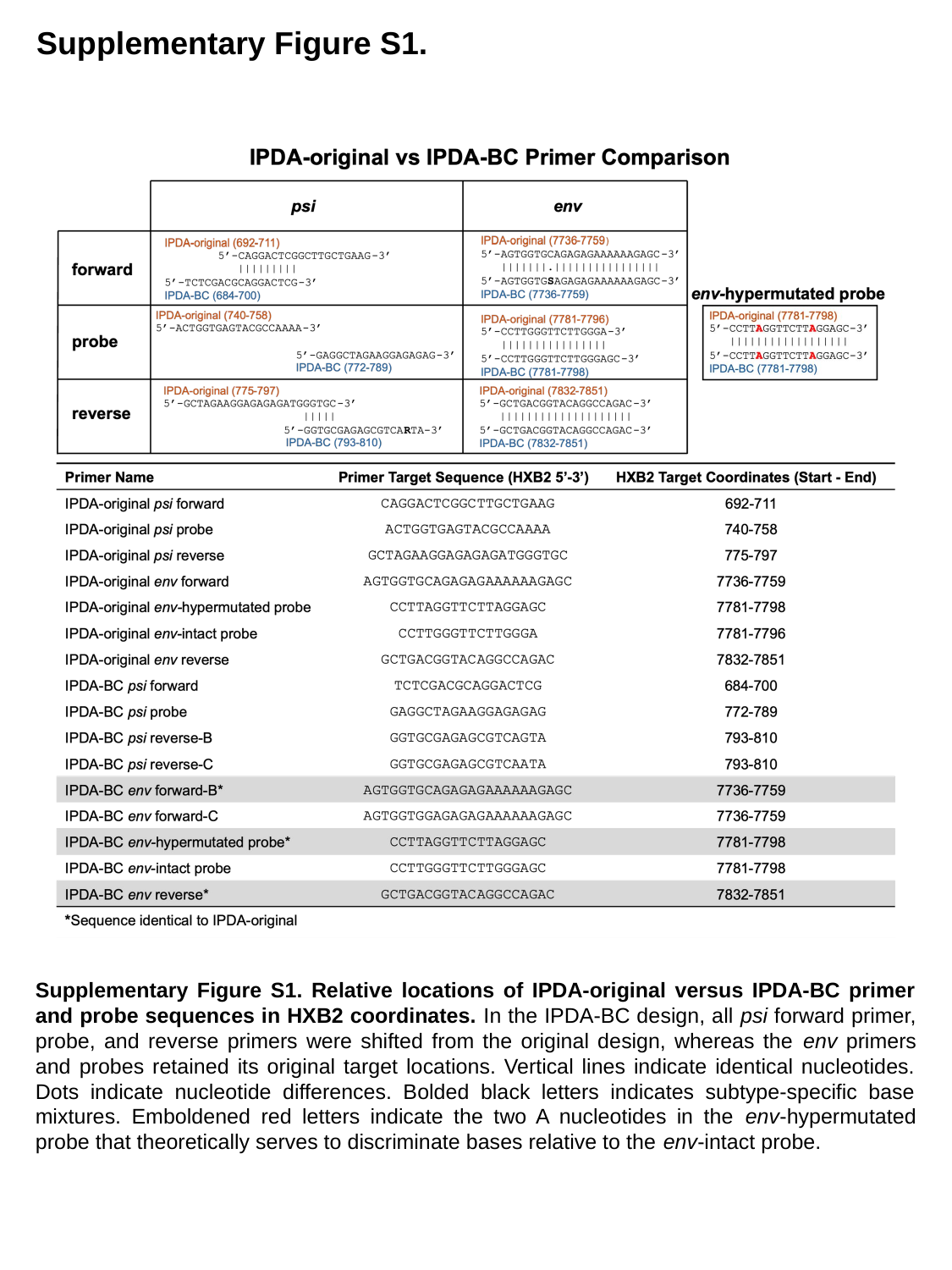

Supplementary Figure S1.
Supplementary Figure S1. Relative locations of IPDA-original versus IPDA-BC primer and probe sequences in HXB2 coordinates. In the IPDA-BC design, all psi forward primer, probe, and reverse primers were shifted from the original design, whereas the env primers and probes retained its original target locations. Vertical lines indicate identical nucleotides. Dots indicate nucleotide differences. Bolded black letters indicates subtype-specific base mixtures. Emboldened red letters indicate the two A nucleotides in the env-hypermutated probe that theoretically serves to discriminate bases relative to the env-intact probe.

## Slide 2
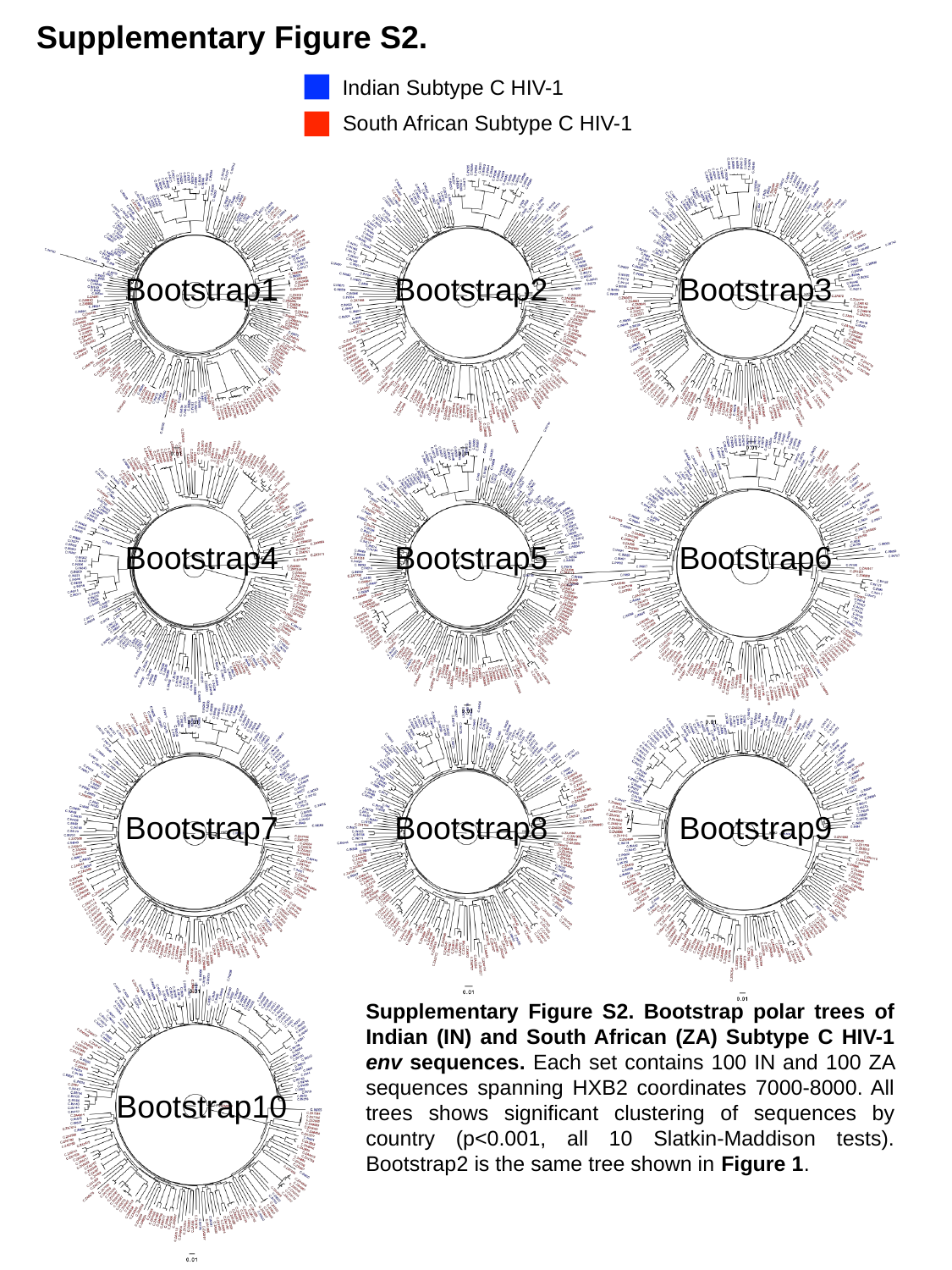

Supplementary Figure S2.
Indian Subtype C HIV-1
South African Subtype C HIV-1
Bootstrap1
Bootstrap2
Bootstrap3
Bootstrap4
Bootstrap5
Bootstrap6
Bootstrap7
Bootstrap8
Bootstrap9
Supplementary Figure S2. Bootstrap polar trees of Indian (IN) and South African (ZA) Subtype C HIV-1 env sequences. Each set contains 100 IN and 100 ZA sequences spanning HXB2 coordinates 7000-8000. All trees shows significant clustering of sequences by country (p<0.001, all 10 Slatkin-Maddison tests). Bootstrap2 is the same tree shown in Figure 1.
Bootstrap10

## Slide 3
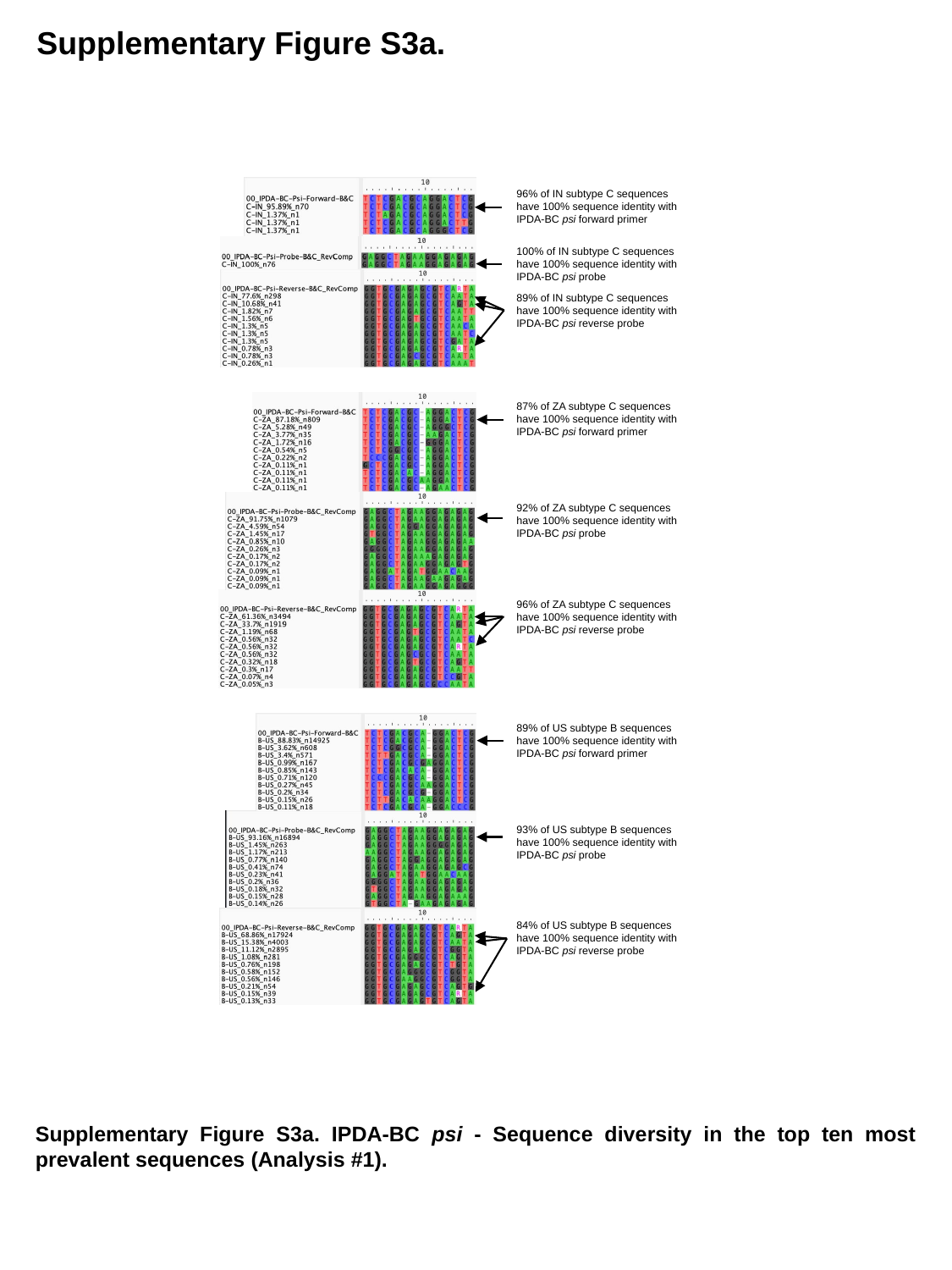

Supplementary Figure S3a.
96% of IN subtype C sequences have 100% sequence identity with IPDA-BC psi forward primer
100% of IN subtype C sequences have 100% sequence identity with IPDA-BC psi probe
89% of IN subtype C sequences have 100% sequence identity with IPDA-BC psi reverse probe
87% of ZA subtype C sequences have 100% sequence identity with IPDA-BC psi forward primer
92% of ZA subtype C sequences have 100% sequence identity with IPDA-BC psi probe
96% of ZA subtype C sequences have 100% sequence identity with IPDA-BC psi reverse probe
89% of US subtype B sequences have 100% sequence identity with IPDA-BC psi forward primer
93% of US subtype B sequences have 100% sequence identity with IPDA-BC psi probe
84% of US subtype B sequences have 100% sequence identity with IPDA-BC psi reverse probe
Supplementary Figure S3a. IPDA-BC psi - Sequence diversity in the top ten most prevalent sequences (Analysis #1).

## Slide 4
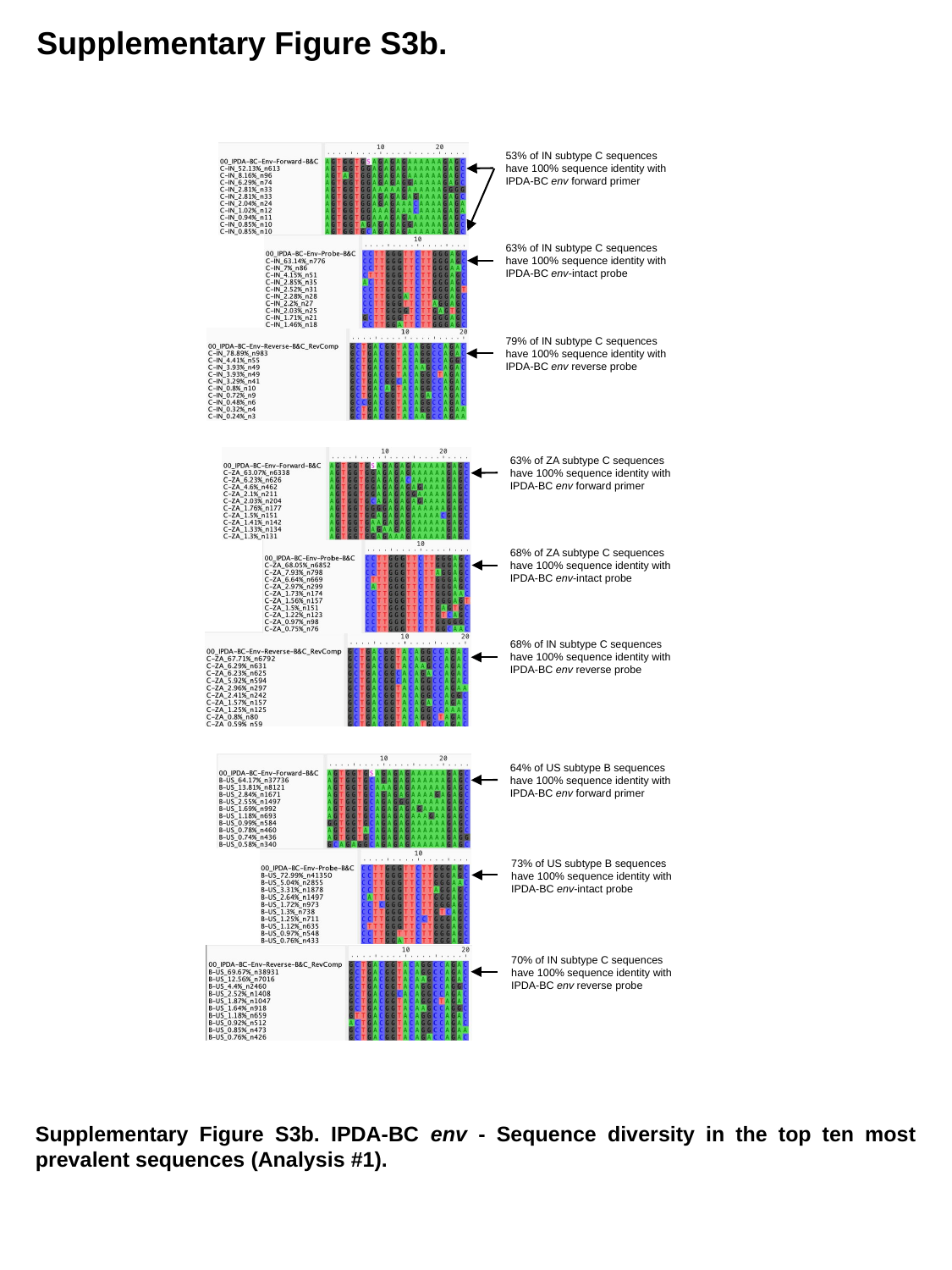

Supplementary Figure S3b.
53% of IN subtype C sequences have 100% sequence identity with IPDA-BC env forward primer
63% of IN subtype C sequences have 100% sequence identity with IPDA-BC env-intact probe
79% of IN subtype C sequences have 100% sequence identity with IPDA-BC env reverse probe
63% of ZA subtype C sequences have 100% sequence identity with IPDA-BC env forward primer
68% of ZA subtype C sequences have 100% sequence identity with IPDA-BC env-intact probe
68% of IN subtype C sequences have 100% sequence identity with IPDA-BC env reverse probe
64% of US subtype B sequences have 100% sequence identity with IPDA-BC env forward primer
73% of US subtype B sequences have 100% sequence identity with IPDA-BC env-intact probe
70% of IN subtype C sequences have 100% sequence identity with IPDA-BC env reverse probe
Supplementary Figure S3b. IPDA-BC env - Sequence diversity in the top ten most prevalent sequences (Analysis #1).

## Slide 5
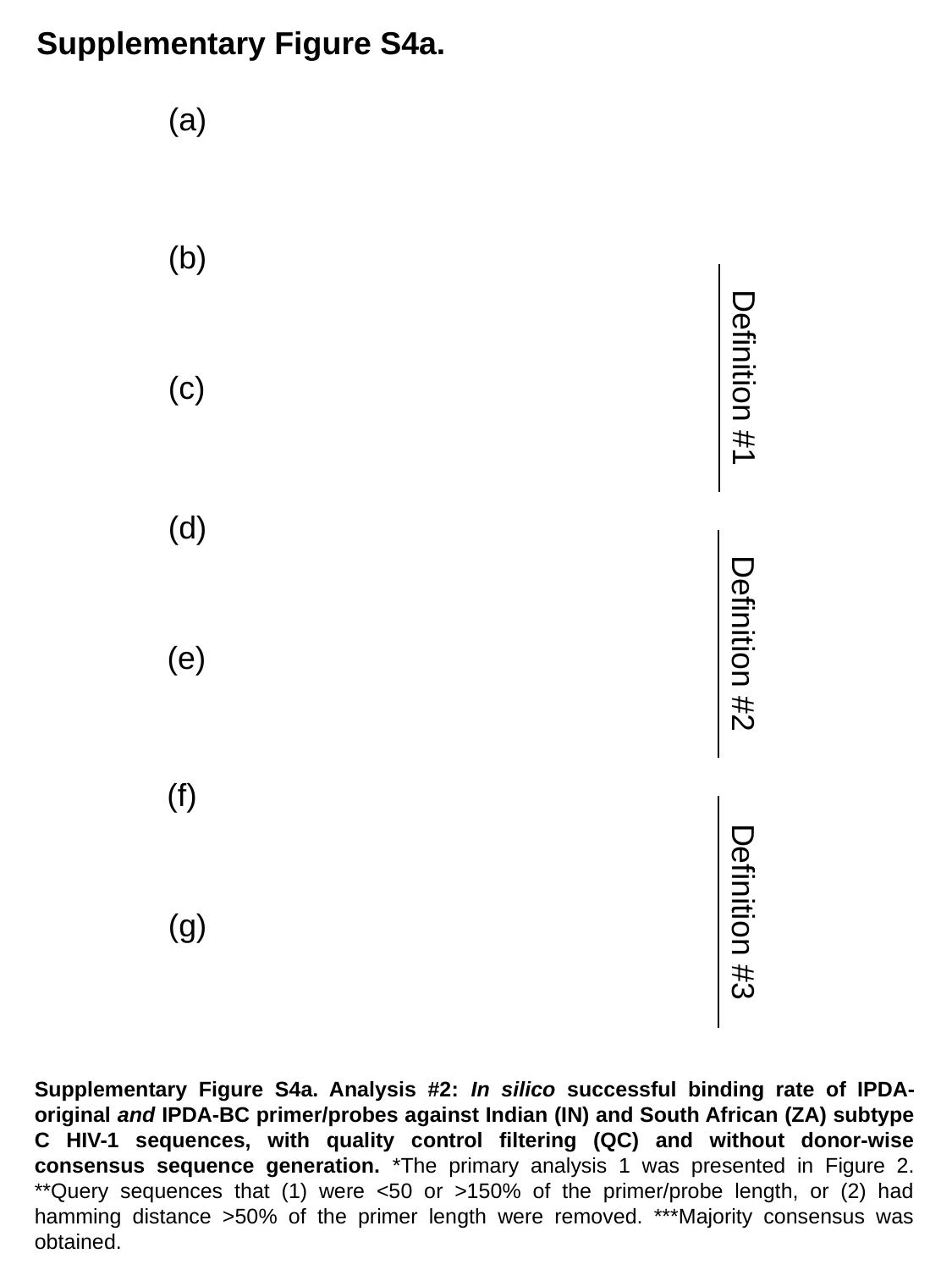

Supplementary Figure S4a.
(a)
(b)
Definition #1
(c)
(d)
Definition #2
(e)
(f)
Definition #3
(g)
Supplementary Figure S4a. Analysis #2: In silico successful binding rate of IPDA-original and IPDA-BC primer/probes against Indian (IN) and South African (ZA) subtype C HIV-1 sequences, with quality control filtering (QC) and without donor-wise consensus sequence generation. *The primary analysis 1 was presented in Figure 2. **Query sequences that (1) were <50 or >150% of the primer/probe length, or (2) had hamming distance >50% of the primer length were removed. ***Majority consensus was obtained.

## Slide 6
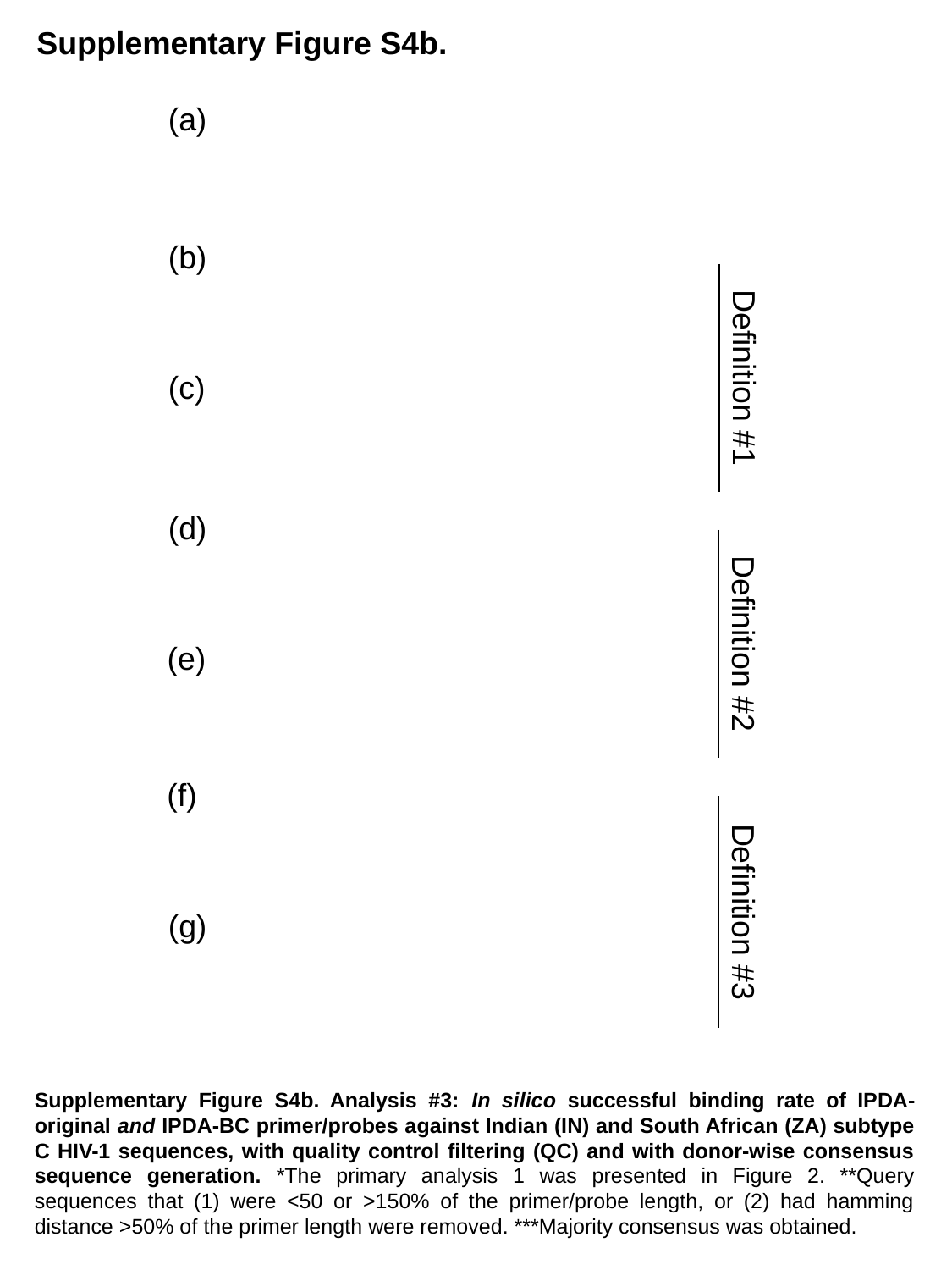

Supplementary Figure S4b.
(a)
(b)
Definition #1
(c)
(d)
Definition #2
(e)
(f)
Definition #3
(g)
Supplementary Figure S4b. Analysis #3: In silico successful binding rate of IPDA-original and IPDA-BC primer/probes against Indian (IN) and South African (ZA) subtype C HIV-1 sequences, with quality control filtering (QC) and with donor-wise consensus sequence generation. *The primary analysis 1 was presented in Figure 2. **Query sequences that (1) were <50 or >150% of the primer/probe length, or (2) had hamming distance >50% of the primer length were removed. ***Majority consensus was obtained.

## Slide 7
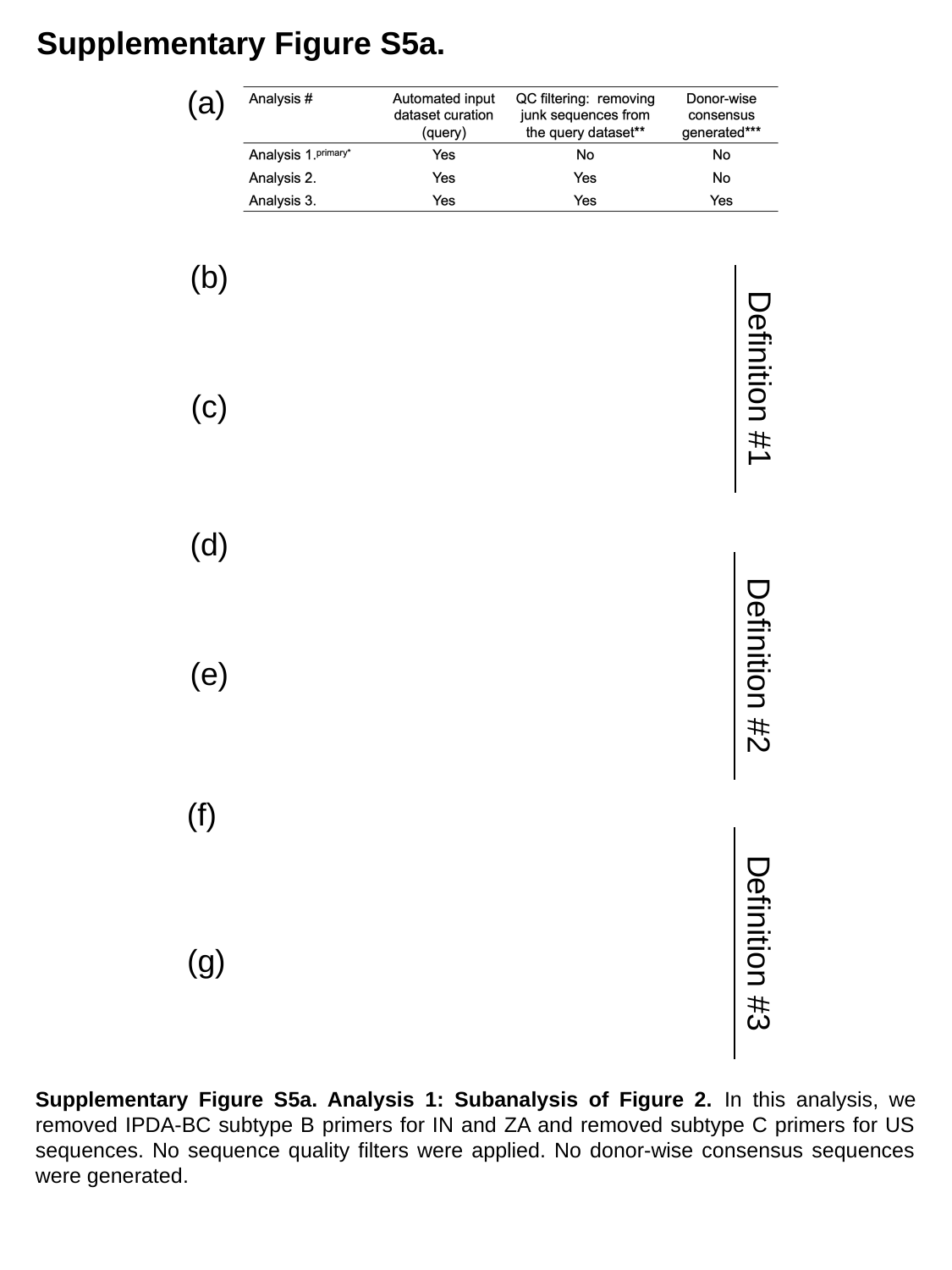

Supplementary Figure S5a.
(a)
(b)
Definition #1
(c)
(d)
Definition #2
(e)
(f)
Definition #3
(g)
Supplementary Figure S5a. Analysis 1: Subanalysis of Figure 2. In this analysis, we removed IPDA-BC subtype B primers for IN and ZA and removed subtype C primers for US sequences. No sequence quality filters were applied. No donor-wise consensus sequences were generated.

## Slide 8
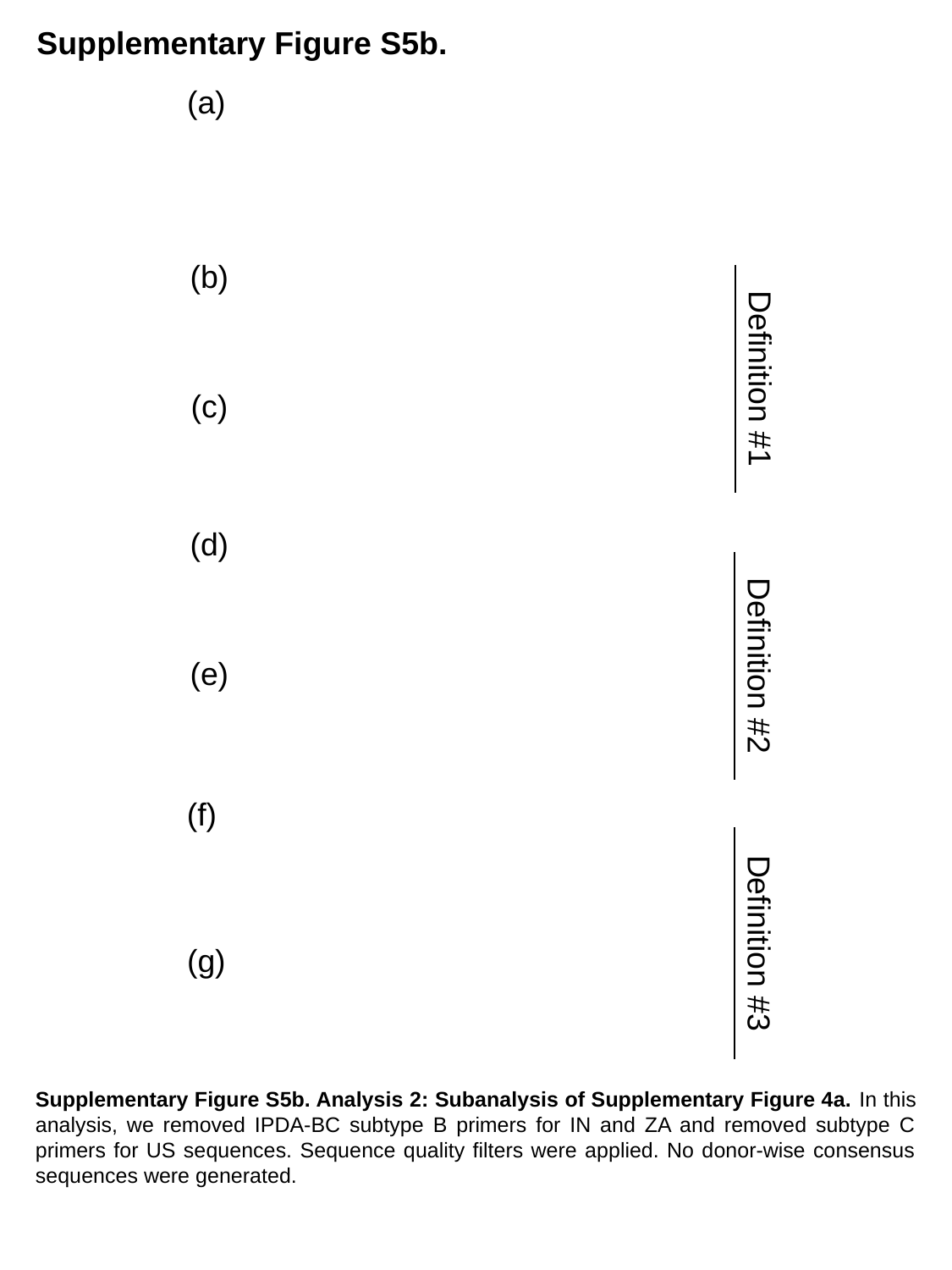

Supplementary Figure S5b.
(a)
(b)
Definition #1
(c)
(d)
Definition #2
(e)
(f)
Definition #3
(g)
Supplementary Figure S5b. Analysis 2: Subanalysis of Supplementary Figure 4a. In this analysis, we removed IPDA-BC subtype B primers for IN and ZA and removed subtype C primers for US sequences. Sequence quality filters were applied. No donor-wise consensus sequences were generated.

## Slide 9
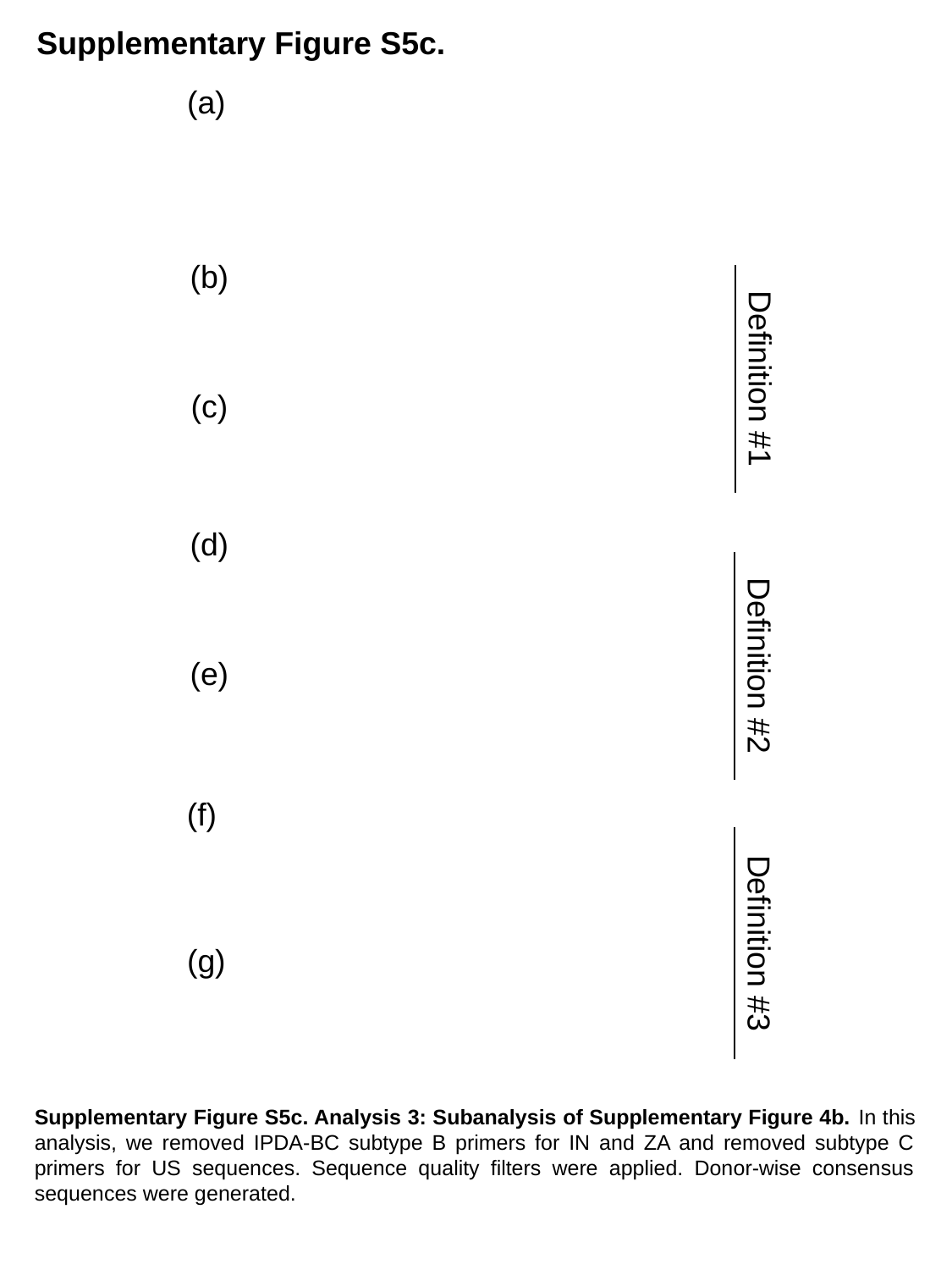

Supplementary Figure S5c.
(a)
(b)
Definition #1
(c)
(d)
Definition #2
(e)
(f)
Definition #3
(g)
Supplementary Figure S5c. Analysis 3: Subanalysis of Supplementary Figure 4b. In this analysis, we removed IPDA-BC subtype B primers for IN and ZA and removed subtype C primers for US sequences. Sequence quality filters were applied. Donor-wise consensus sequences were generated.

## Slide 10
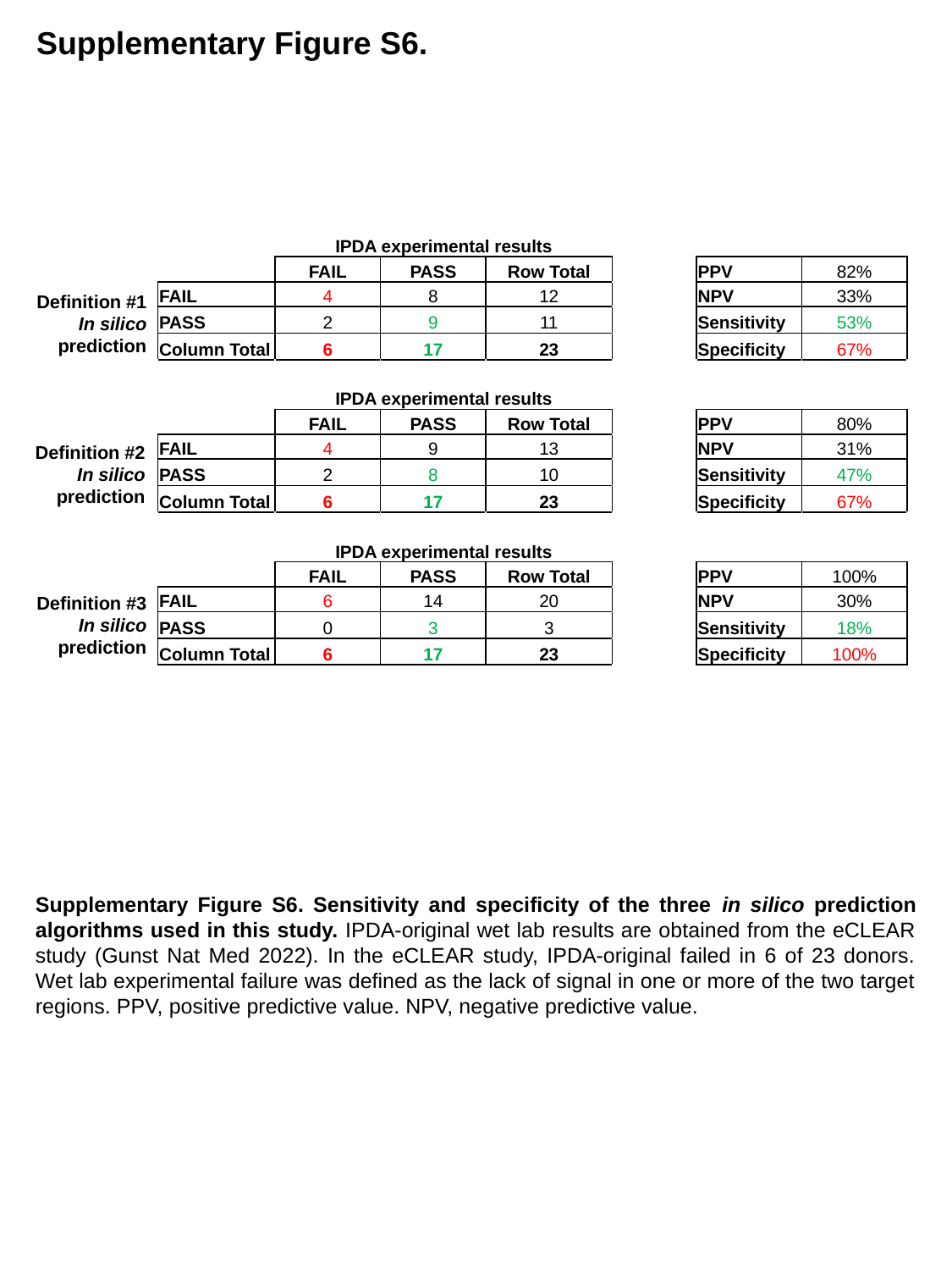

Supplementary Figure S6.
| | IPDA experimental results | | | | | |
| --- | --- | --- | --- | --- | --- | --- |
| | FAIL | PASS | Row Total | | PPV | 82% |
| FAIL | 4 | 8 | 12 | | NPV | 33% |
| PASS | 2 | 9 | 11 | | Sensitivity | 53% |
| Column Total | 6 | 17 | 23 | | Specificity | 67% |
| | | | | | | |
| | IPDA experimental results | | | | | |
| | FAIL | PASS | Row Total | | PPV | 80% |
| FAIL | 4 | 9 | 13 | | NPV | 31% |
| PASS | 2 | 8 | 10 | | Sensitivity | 47% |
| Column Total | 6 | 17 | 23 | | Specificity | 67% |
| | | | | | | |
| | IPDA experimental results | | | | | |
| | FAIL | PASS | Row Total | | PPV | 100% |
| FAIL | 6 | 14 | 20 | | NPV | 30% |
| PASS | 0 | 3 | 3 | | Sensitivity | 18% |
| Column Total | 6 | 17 | 23 | | Specificity | 100% |
Definition #1 In silico prediction
Definition #2 In silico prediction
Definition #3 In silico prediction
Supplementary Figure S6. Sensitivity and specificity of the three in silico prediction algorithms used in this study. IPDA-original wet lab results are obtained from the eCLEAR study (Gunst Nat Med 2022). In the eCLEAR study, IPDA-original failed in 6 of 23 donors. Wet lab experimental failure was defined as the lack of signal in one or more of the two target regions. PPV, positive predictive value. NPV, negative predictive value.

## Slide 11
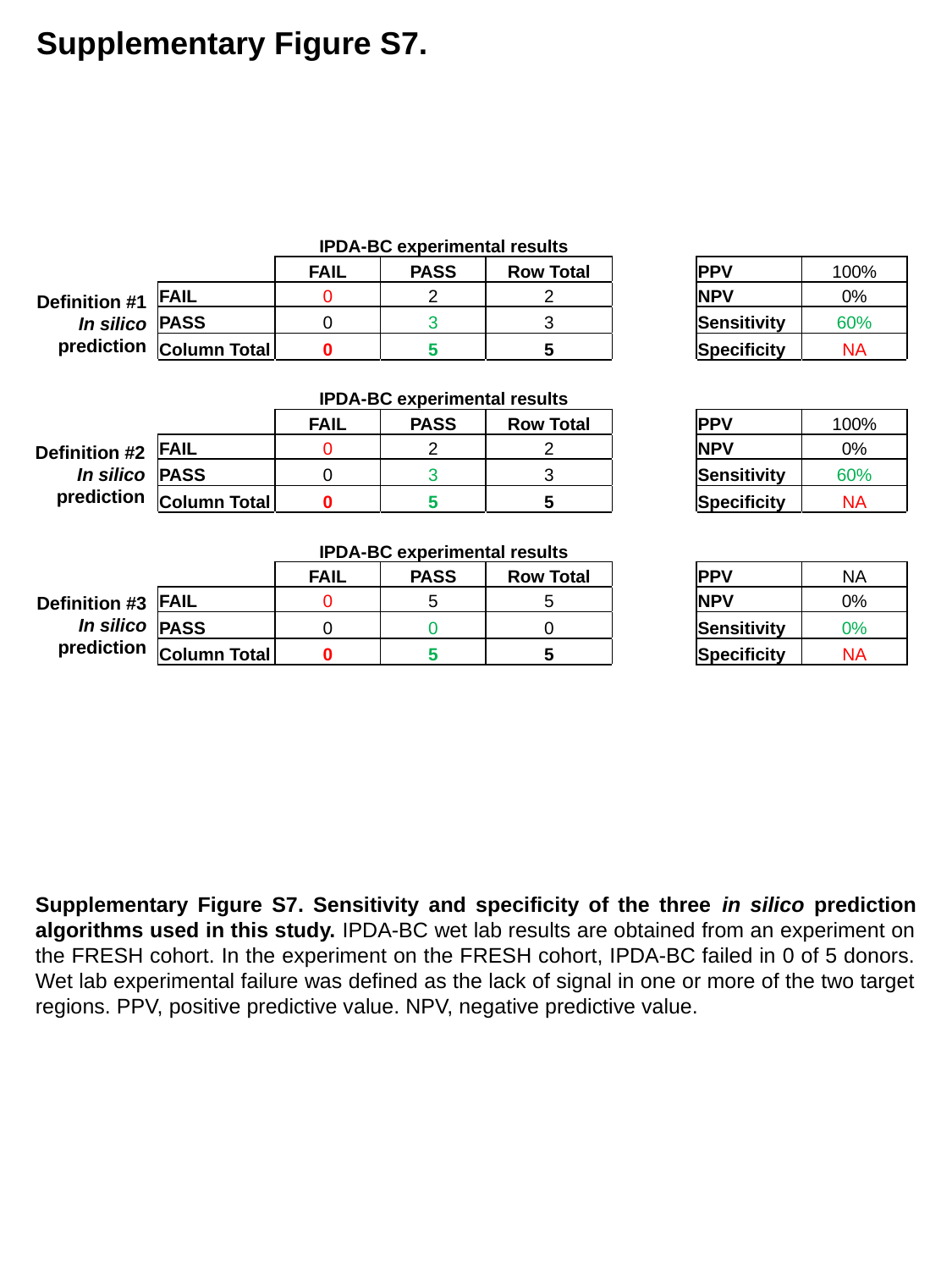

Supplementary Figure S7.
| | IPDA-BC experimental results | | | | | |
| --- | --- | --- | --- | --- | --- | --- |
| | FAIL | PASS | Row Total | | PPV | 100% |
| FAIL | 0 | 2 | 2 | | NPV | 0% |
| PASS | 0 | 3 | 3 | | Sensitivity | 60% |
| Column Total | 0 | 5 | 5 | | Specificity | NA |
| | | | | | | |
| | IPDA-BC experimental results | | | | | |
| | FAIL | PASS | Row Total | | PPV | 100% |
| FAIL | 0 | 2 | 2 | | NPV | 0% |
| PASS | 0 | 3 | 3 | | Sensitivity | 60% |
| Column Total | 0 | 5 | 5 | | Specificity | NA |
| | | | | | | |
| | IPDA-BC experimental results | | | | | |
| | FAIL | PASS | Row Total | | PPV | NA |
| FAIL | 0 | 5 | 5 | | NPV | 0% |
| PASS | 0 | 0 | 0 | | Sensitivity | 0% |
| Column Total | 0 | 5 | 5 | | Specificity | NA |
Definition #1 In silico prediction
Definition #2 In silico prediction
Definition #3 In silico prediction
Supplementary Figure S7. Sensitivity and specificity of the three in silico prediction algorithms used in this study. IPDA-BC wet lab results are obtained from an experiment on the FRESH cohort. In the experiment on the FRESH cohort, IPDA-BC failed in 0 of 5 donors. Wet lab experimental failure was defined as the lack of signal in one or more of the two target regions. PPV, positive predictive value. NPV, negative predictive value.

## Slide 12
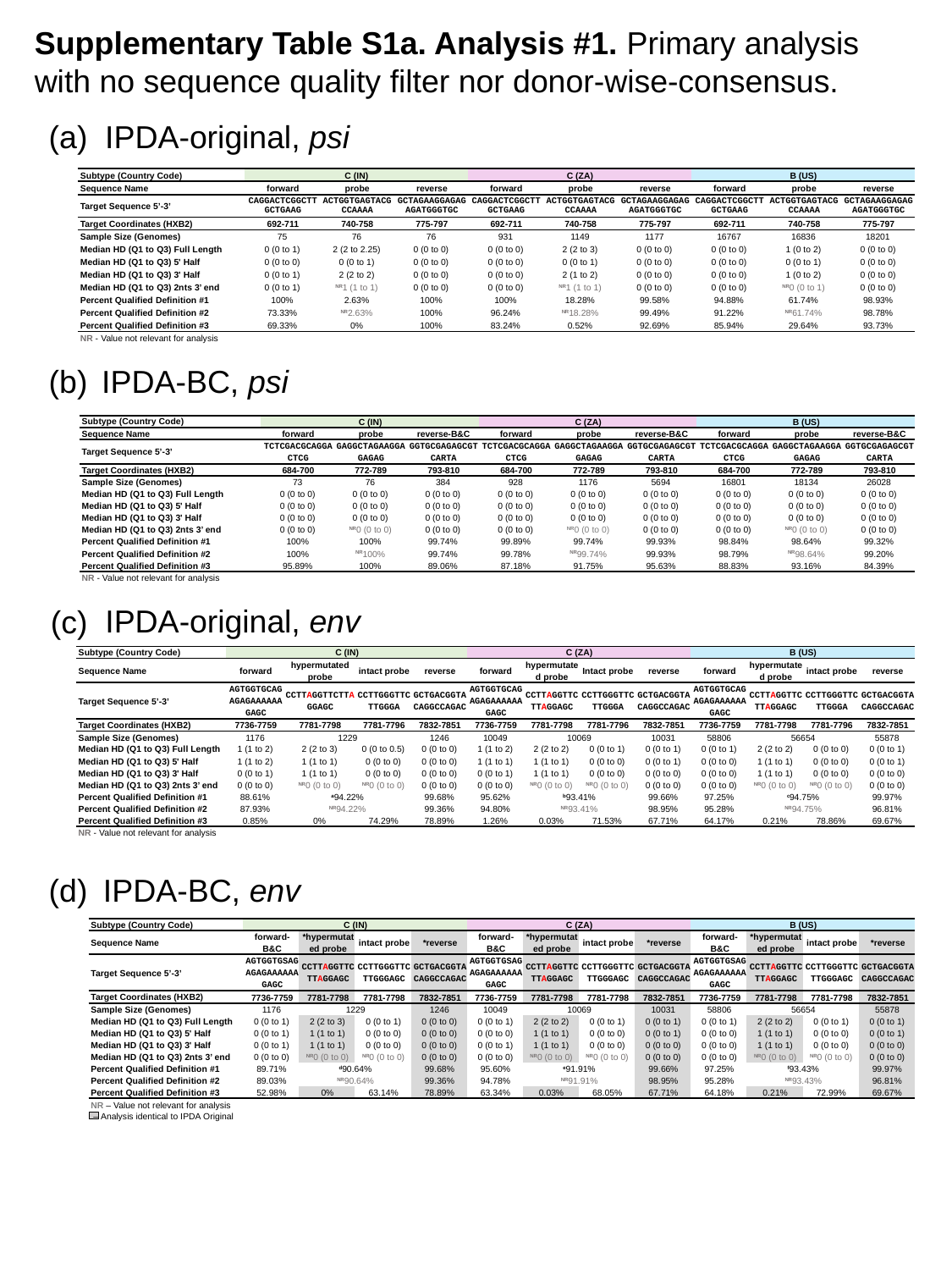

Supplementary Table S1a. Analysis #1. Primary analysis with no sequence quality filter nor donor-wise-consensus.
(a)
IPDA-original, psi
| Subtype (Country Code) | C (IN) | | | C (ZA) | | | B (US) | | |
| --- | --- | --- | --- | --- | --- | --- | --- | --- | --- |
| Sequence Name | forward | probe | reverse | forward | probe | reverse | forward | probe | reverse |
| Target Sequence 5’-3’ | CAGGACTCGGCTTGCTGAAG | ACTGGTGAGTACGCCAAAA | GCTAGAAGGAGAGAGATGGGTGC | CAGGACTCGGCTTGCTGAAG | ACTGGTGAGTACGCCAAAA | GCTAGAAGGAGAGAGATGGGTGC | CAGGACTCGGCTTGCTGAAG | ACTGGTGAGTACGCCAAAA | GCTAGAAGGAGAGAGATGGGTGC |
| Target Coordinates (HXB2) | 692-711 | 740-758 | 775-797 | 692-711 | 740-758 | 775-797 | 692-711 | 740-758 | 775-797 |
| Sample Size (Genomes) | 75 | 76 | 76 | 931 | 1149 | 1177 | 16767 | 16836 | 18201 |
| Median HD (Q1 to Q3) Full Length | 0 (0 to 1) | 2 (2 to 2.25) | 0 (0 to 0) | 0 (0 to 0) | 2 (2 to 3) | 0 (0 to 0) | 0 (0 to 0) | 1 (0 to 2) | 0 (0 to 0) |
| Median HD (Q1 to Q3) 5' Half | 0 (0 to 0) | 0 (0 to 1) | 0 (0 to 0) | 0 (0 to 0) | 0 (0 to 1) | 0 (0 to 0) | 0 (0 to 0) | 0 (0 to 1) | 0 (0 to 0) |
| Median HD (Q1 to Q3) 3' Half | 0 (0 to 1) | 2 (2 to 2) | 0 (0 to 0) | 0 (0 to 0) | 2 (1 to 2) | 0 (0 to 0) | 0 (0 to 0) | 1 (0 to 2) | 0 (0 to 0) |
| Median HD (Q1 to Q3) 2nts 3’ end | 0 (0 to 1) | NR1 (1 to 1) | 0 (0 to 0) | 0 (0 to 0) | NR1 (1 to 1) | 0 (0 to 0) | 0 (0 to 0) | NR0 (0 to 1) | 0 (0 to 0) |
| Percent Qualified Definition #1 | 100% | 2.63% | 100% | 100% | 18.28% | 99.58% | 94.88% | 61.74% | 98.93% |
| Percent Qualified Definition #2 | 73.33% | NR2.63% | 100% | 96.24% | NR18.28% | 99.49% | 91.22% | NR61.74% | 98.78% |
| Percent Qualified Definition #3 | 69.33% | 0% | 100% | 83.24% | 0.52% | 92.69% | 85.94% | 29.64% | 93.73% |
| NR - Value not relevant for analysis | | | | | | | | | |
IPDA-BC, psi
(b)
| Subtype (Country Code) | C (IN) | | | C (ZA) | | | B (US) | | |
| --- | --- | --- | --- | --- | --- | --- | --- | --- | --- |
| Sequence Name | forward | probe | reverse-B&C | forward | probe | reverse-B&C | forward | probe | reverse-B&C |
| Target Sequence 5’-3’ | TCTCGACGCAGGACTCG | GAGGCTAGAAGGAGAGAG | GGTGCGAGAGCGTCARTA | TCTCGACGCAGGACTCG | GAGGCTAGAAGGAGAGAG | GGTGCGAGAGCGTCARTA | TCTCGACGCAGGACTCG | GAGGCTAGAAGGAGAGAG | GGTGCGAGAGCGTCARTA |
| Target Coordinates (HXB2) | 684-700 | 772-789 | 793-810 | 684-700 | 772-789 | 793-810 | 684-700 | 772-789 | 793-810 |
| Sample Size (Genomes) | 73 | 76 | 384 | 928 | 1176 | 5694 | 16801 | 18134 | 26028 |
| Median HD (Q1 to Q3) Full Length | 0 (0 to 0) | 0 (0 to 0) | 0 (0 to 0) | 0 (0 to 0) | 0 (0 to 0) | 0 (0 to 0) | 0 (0 to 0) | 0 (0 to 0) | 0 (0 to 0) |
| Median HD (Q1 to Q3) 5' Half | 0 (0 to 0) | 0 (0 to 0) | 0 (0 to 0) | 0 (0 to 0) | 0 (0 to 0) | 0 (0 to 0) | 0 (0 to 0) | 0 (0 to 0) | 0 (0 to 0) |
| Median HD (Q1 to Q3) 3' Half | 0 (0 to 0) | 0 (0 to 0) | 0 (0 to 0) | 0 (0 to 0) | 0 (0 to 0) | 0 (0 to 0) | 0 (0 to 0) | 0 (0 to 0) | 0 (0 to 0) |
| Median HD (Q1 to Q3) 2nts 3’ end | 0 (0 to 0) | NR0 (0 to 0) | 0 (0 to 0) | 0 (0 to 0) | NR0 (0 to 0) | 0 (0 to 0) | 0 (0 to 0) | NR0 (0 to 0) | 0 (0 to 0) |
| Percent Qualified Definition #1 | 100% | 100% | 99.74% | 99.89% | 99.74% | 99.93% | 98.84% | 98.64% | 99.32% |
| Percent Qualified Definition #2 | 100% | NR100% | 99.74% | 99.78% | NR99.74% | 99.93% | 98.79% | NR98.64% | 99.20% |
| Percent Qualified Definition #3 | 95.89% | 100% | 89.06% | 87.18% | 91.75% | 95.63% | 88.83% | 93.16% | 84.39% |
| NR - Value not relevant for analysis | | | | | | | | | |
IPDA-original, env
(c)
| Subtype (Country Code) | C (IN) | | | | C (ZA) | | | | B (US) | | | |
| --- | --- | --- | --- | --- | --- | --- | --- | --- | --- | --- | --- | --- |
| Sequence Name | forward | hypermutated probe | intact probe | reverse | forward | hypermutated probe | Intact probe | reverse | forward | hypermutated probe | intact probe | reverse |
| Target Sequence 5’-3’ | AGTGGTGCAGAGAGAAAAAAGAGC | CCTTAGGTTCTTAGGAGC | CCTTGGGTTCTTGGGA | GCTGACGGTACAGGCCAGAC | AGTGGTGCAGAGAGAAAAAAGAGC | CCTTAGGTTCTTAGGAGC | CCTTGGGTTCTTGGGA | GCTGACGGTACAGGCCAGAC | AGTGGTGCAGAGAGAAAAAAGAGC | CCTTAGGTTCTTAGGAGC | CCTTGGGTTCTTGGGA | GCTGACGGTACAGGCCAGAC |
| Target Coordinates (HXB2) | 7736-7759 | 7781-7798 | 7781-7796 | 7832-7851 | 7736-7759 | 7781-7798 | 7781-7796 | 7832-7851 | 7736-7759 | 7781-7798 | 7781-7796 | 7832-7851 |
| Sample Size (Genomes) | 1176 | 1229 | | 1246 | 10049 | 10069 | | 10031 | 58806 | 56654 | | 55878 |
| Median HD (Q1 to Q3) Full Length | 1 (1 to 2) | 2 (2 to 3) | 0 (0 to 0.5) | 0 (0 to 0) | 1 (1 to 2) | 2 (2 to 2) | 0 (0 to 1) | 0 (0 to 1) | 0 (0 to 1) | 2 (2 to 2) | 0 (0 to 0) | 0 (0 to 1) |
| Median HD (Q1 to Q3) 5' Half | 1 (1 to 2) | 1 (1 to 1) | 0 (0 to 0) | 0 (0 to 0) | 1 (1 to 1) | 1 (1 to 1) | 0 (0 to 0) | 0 (0 to 1) | 0 (0 to 0) | 1 (1 to 1) | 0 (0 to 0) | 0 (0 to 1) |
| Median HD (Q1 to Q3) 3' Half | 0 (0 to 1) | 1 (1 to 1) | 0 (0 to 0) | 0 (0 to 0) | 0 (0 to 1) | 1 (1 to 1) | 0 (0 to 0) | 0 (0 to 0) | 0 (0 to 0) | 1 (1 to 1) | 0 (0 to 0) | 0 (0 to 0) |
| Median HD (Q1 to Q3) 2nts 3’ end | 0 (0 to 0) | NR0 (0 to 0) | NR0 (0 to 0) | 0 (0 to 0) | 0 (0 to 0) | NR0 (0 to 0) | NR0 (0 to 0) | 0 (0 to 0) | 0 (0 to 0) | NR0 (0 to 0) | NR0 (0 to 0) | 0 (0 to 0) |
| Percent Qualified Definition #1 | 88.61% | a94.22% | | 99.68% | 95.62% | b93.41% | | 99.66% | 97.25% | c94.75% | | 99.97% |
| Percent Qualified Definition #2 | 87.93% | NR94.22% | | 99.36% | 94.80% | NR93.41% | | 98.95% | 95.28% | NR94.75% | | 96.81% |
| Percent Qualified Definition #3 | 0.85% | 0% | 74.29% | 78.89% | 1.26% | 0.03% | 71.53% | 67.71% | 64.17% | 0.21% | 78.86% | 69.67% |
| NR - Value not relevant for analysis | | | | | | | | | | | | |
(d)
IPDA-BC, env
| Subtype (Country Code) | C (IN) | | | | C (ZA) | | | | B (US) | | | |
| --- | --- | --- | --- | --- | --- | --- | --- | --- | --- | --- | --- | --- |
| Sequence Name | forward-B&C | \*hypermutated probe | intact probe | \*reverse | forward-B&C | \*hypermutated probe | intact probe | \*reverse | forward-B&C | \*hypermutated probe | intact probe | \*reverse |
| Target Sequence 5’-3’ | AGTGGTGSAGAGAGAAAAAAGAGC | CCTTAGGTTCTTAGGAGC | CCTTGGGTTCTTGGGAGC | GCTGACGGTACAGGCCAGAC | AGTGGTGSAGAGAGAAAAAAGAGC | CCTTAGGTTCTTAGGAGC | CCTTGGGTTCTTGGGAGC | GCTGACGGTACAGGCCAGAC | AGTGGTGSAGAGAGAAAAAAGAGC | CCTTAGGTTCTTAGGAGC | CCTTGGGTTCTTGGGAGC | GCTGACGGTACAGGCCAGAC |
| Target Coordinates (HXB2) | 7736-7759 | 7781-7798 | 7781-7798 | 7832-7851 | 7736-7759 | 7781-7798 | 7781-7798 | 7832-7851 | 7736-7759 | 7781-7798 | 7781-7798 | 7832-7851 |
| Sample Size (Genomes) | 1176 | 1229 | | 1246 | 10049 | 10069 | | 10031 | 58806 | 56654 | | 55878 |
| Median HD (Q1 to Q3) Full Length | 0 (0 to 1) | 2 (2 to 3) | 0 (0 to 1) | 0 (0 to 0) | 0 (0 to 1) | 2 (2 to 2) | 0 (0 to 1) | 0 (0 to 1) | 0 (0 to 1) | 2 (2 to 2) | 0 (0 to 1) | 0 (0 to 1) |
| Median HD (Q1 to Q3) 5' Half | 0 (0 to 1) | 1 (1 to 1) | 0 (0 to 0) | 0 (0 to 0) | 0 (0 to 0) | 1 (1 to 1) | 0 (0 to 0) | 0 (0 to 1) | 0 (0 to 0) | 1 (1 to 1) | 0 (0 to 0) | 0 (0 to 1) |
| Median HD (Q1 to Q3) 3' Half | 0 (0 to 1) | 1 (1 to 1) | 0 (0 to 0) | 0 (0 to 0) | 0 (0 to 1) | 1 (1 to 1) | 0 (0 to 0) | 0 (0 to 0) | 0 (0 to 0) | 1 (1 to 1) | 0 (0 to 0) | 0 (0 to 0) |
| Median HD (Q1 to Q3) 2nts 3’ end | 0 (0 to 0) | NR0 (0 to 0) | NR0 (0 to 0) | 0 (0 to 0) | 0 (0 to 0) | NR0 (0 to 0) | NR0 (0 to 0) | 0 (0 to 0) | 0 (0 to 0) | NR0 (0 to 0) | NR0 (0 to 0) | 0 (0 to 0) |
| Percent Qualified Definition #1 | 89.71% | d90.64% | | 99.68% | 95.60% | e91.91% | | 99.66% | 97.25% | f93.43% | | 99.97% |
| Percent Qualified Definition #2 | 89.03% | NR90.64% | | 99.36% | 94.78% | NR91.91% | | 98.95% | 95.28% | NR93.43% | | 96.81% |
| Percent Qualified Definition #3 | 52.98% | 0% | 63.14% | 78.89% | 63.34% | 0.03% | 68.05% | 67.71% | 64.18% | 0.21% | 72.99% | 69.67% |
| NR – Value not relevant for analysis – Analysis identical to IPDA Original | | | | | | | | | | | | |

## Slide 13
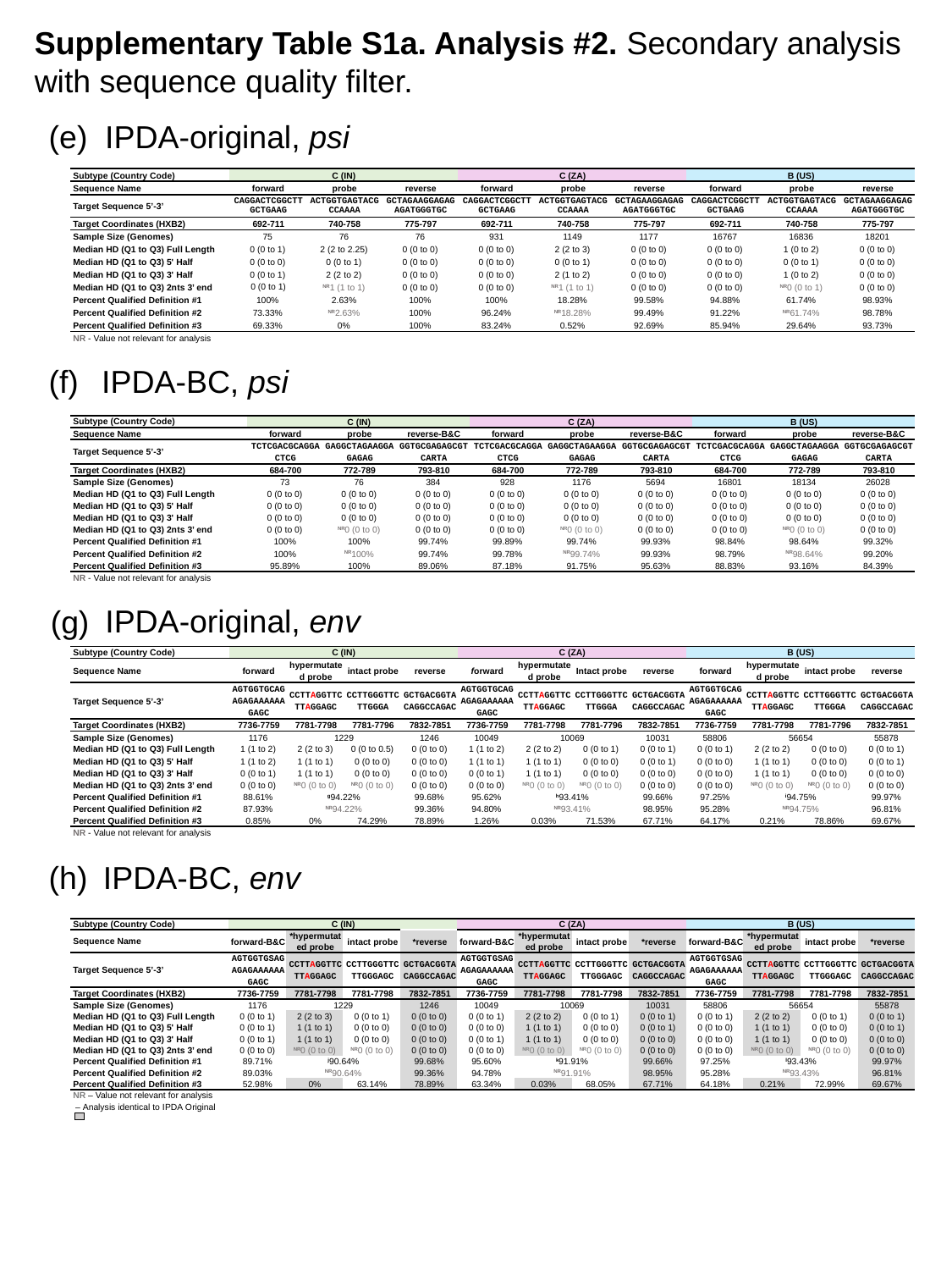

Supplementary Table S1a. Analysis #2. Secondary analysis with sequence quality filter.
(e)
IPDA-original, psi
| Subtype (Country Code) | C (IN) | | | C (ZA) | | | B (US) | | |
| --- | --- | --- | --- | --- | --- | --- | --- | --- | --- |
| Sequence Name | forward | probe | reverse | forward | probe | reverse | forward | probe | reverse |
| Target Sequence 5’-3’ | CAGGACTCGGCTTGCTGAAG | ACTGGTGAGTACGCCAAAA | GCTAGAAGGAGAGAGATGGGTGC | CAGGACTCGGCTTGCTGAAG | ACTGGTGAGTACGCCAAAA | GCTAGAAGGAGAGAGATGGGTGC | CAGGACTCGGCTTGCTGAAG | ACTGGTGAGTACGCCAAAA | GCTAGAAGGAGAGAGATGGGTGC |
| Target Coordinates (HXB2) | 692-711 | 740-758 | 775-797 | 692-711 | 740-758 | 775-797 | 692-711 | 740-758 | 775-797 |
| Sample Size (Genomes) | 75 | 76 | 76 | 931 | 1149 | 1177 | 16767 | 16836 | 18201 |
| Median HD (Q1 to Q3) Full Length | 0 (0 to 1) | 2 (2 to 2.25) | 0 (0 to 0) | 0 (0 to 0) | 2 (2 to 3) | 0 (0 to 0) | 0 (0 to 0) | 1 (0 to 2) | 0 (0 to 0) |
| Median HD (Q1 to Q3) 5' Half | 0 (0 to 0) | 0 (0 to 1) | 0 (0 to 0) | 0 (0 to 0) | 0 (0 to 1) | 0 (0 to 0) | 0 (0 to 0) | 0 (0 to 1) | 0 (0 to 0) |
| Median HD (Q1 to Q3) 3' Half | 0 (0 to 1) | 2 (2 to 2) | 0 (0 to 0) | 0 (0 to 0) | 2 (1 to 2) | 0 (0 to 0) | 0 (0 to 0) | 1 (0 to 2) | 0 (0 to 0) |
| Median HD (Q1 to Q3) 2nts 3’ end | 0 (0 to 1) | NR1 (1 to 1) | 0 (0 to 0) | 0 (0 to 0) | NR1 (1 to 1) | 0 (0 to 0) | 0 (0 to 0) | NR0 (0 to 1) | 0 (0 to 0) |
| Percent Qualified Definition #1 | 100% | 2.63% | 100% | 100% | 18.28% | 99.58% | 94.88% | 61.74% | 98.93% |
| Percent Qualified Definition #2 | 73.33% | NR2.63% | 100% | 96.24% | NR18.28% | 99.49% | 91.22% | NR61.74% | 98.78% |
| Percent Qualified Definition #3 | 69.33% | 0% | 100% | 83.24% | 0.52% | 92.69% | 85.94% | 29.64% | 93.73% |
| NR - Value not relevant for analysis | | | | | | | | | |
IPDA-BC, psi
(f)
| Subtype (Country Code) | C (IN) | | | C (ZA) | | | B (US) | | |
| --- | --- | --- | --- | --- | --- | --- | --- | --- | --- |
| Sequence Name | forward | probe | reverse-B&C | forward | probe | reverse-B&C | forward | probe | reverse-B&C |
| Target Sequence 5’-3’ | TCTCGACGCAGGACTCG | GAGGCTAGAAGGAGAGAG | GGTGCGAGAGCGTCARTA | TCTCGACGCAGGACTCG | GAGGCTAGAAGGAGAGAG | GGTGCGAGAGCGTCARTA | TCTCGACGCAGGACTCG | GAGGCTAGAAGGAGAGAG | GGTGCGAGAGCGTCARTA |
| Target Coordinates (HXB2) | 684-700 | 772-789 | 793-810 | 684-700 | 772-789 | 793-810 | 684-700 | 772-789 | 793-810 |
| Sample Size (Genomes) | 73 | 76 | 384 | 928 | 1176 | 5694 | 16801 | 18134 | 26028 |
| Median HD (Q1 to Q3) Full Length | 0 (0 to 0) | 0 (0 to 0) | 0 (0 to 0) | 0 (0 to 0) | 0 (0 to 0) | 0 (0 to 0) | 0 (0 to 0) | 0 (0 to 0) | 0 (0 to 0) |
| Median HD (Q1 to Q3) 5' Half | 0 (0 to 0) | 0 (0 to 0) | 0 (0 to 0) | 0 (0 to 0) | 0 (0 to 0) | 0 (0 to 0) | 0 (0 to 0) | 0 (0 to 0) | 0 (0 to 0) |
| Median HD (Q1 to Q3) 3' Half | 0 (0 to 0) | 0 (0 to 0) | 0 (0 to 0) | 0 (0 to 0) | 0 (0 to 0) | 0 (0 to 0) | 0 (0 to 0) | 0 (0 to 0) | 0 (0 to 0) |
| Median HD (Q1 to Q3) 2nts 3’ end | 0 (0 to 0) | NR0 (0 to 0) | 0 (0 to 0) | 0 (0 to 0) | NR0 (0 to 0) | 0 (0 to 0) | 0 (0 to 0) | NR0 (0 to 0) | 0 (0 to 0) |
| Percent Qualified Definition #1 | 100% | 100% | 99.74% | 99.89% | 99.74% | 99.93% | 98.84% | 98.64% | 99.32% |
| Percent Qualified Definition #2 | 100% | NR100% | 99.74% | 99.78% | NR99.74% | 99.93% | 98.79% | NR98.64% | 99.20% |
| Percent Qualified Definition #3 | 95.89% | 100% | 89.06% | 87.18% | 91.75% | 95.63% | 88.83% | 93.16% | 84.39% |
| NR - Value not relevant for analysis | | | | | | | | | |
IPDA-original, env
(g)
| Subtype (Country Code) | C (IN) | | | | C (ZA) | | | | B (US) | | | |
| --- | --- | --- | --- | --- | --- | --- | --- | --- | --- | --- | --- | --- |
| Sequence Name | forward | hypermutated probe | intact probe | reverse | forward | hypermutated probe | Intact probe | reverse | forward | hypermutated probe | intact probe | reverse |
| Target Sequence 5’-3’ | AGTGGTGCAGAGAGAAAAAAGAGC | CCTTAGGTTCTTAGGAGC | CCTTGGGTTCTTGGGA | GCTGACGGTACAGGCCAGAC | AGTGGTGCAGAGAGAAAAAAGAGC | CCTTAGGTTCTTAGGAGC | CCTTGGGTTCTTGGGA | GCTGACGGTACAGGCCAGAC | AGTGGTGCAGAGAGAAAAAAGAGC | CCTTAGGTTCTTAGGAGC | CCTTGGGTTCTTGGGA | GCTGACGGTACAGGCCAGAC |
| Target Coordinates (HXB2) | 7736-7759 | 7781-7798 | 7781-7796 | 7832-7851 | 7736-7759 | 7781-7798 | 7781-7796 | 7832-7851 | 7736-7759 | 7781-7798 | 7781-7796 | 7832-7851 |
| Sample Size (Genomes) | 1176 | 1229 | | 1246 | 10049 | 10069 | | 10031 | 58806 | 56654 | | 55878 |
| Median HD (Q1 to Q3) Full Length | 1 (1 to 2) | 2 (2 to 3) | 0 (0 to 0.5) | 0 (0 to 0) | 1 (1 to 2) | 2 (2 to 2) | 0 (0 to 1) | 0 (0 to 1) | 0 (0 to 1) | 2 (2 to 2) | 0 (0 to 0) | 0 (0 to 1) |
| Median HD (Q1 to Q3) 5' Half | 1 (1 to 2) | 1 (1 to 1) | 0 (0 to 0) | 0 (0 to 0) | 1 (1 to 1) | 1 (1 to 1) | 0 (0 to 0) | 0 (0 to 1) | 0 (0 to 0) | 1 (1 to 1) | 0 (0 to 0) | 0 (0 to 1) |
| Median HD (Q1 to Q3) 3' Half | 0 (0 to 1) | 1 (1 to 1) | 0 (0 to 0) | 0 (0 to 0) | 0 (0 to 1) | 1 (1 to 1) | 0 (0 to 0) | 0 (0 to 0) | 0 (0 to 0) | 1 (1 to 1) | 0 (0 to 0) | 0 (0 to 0) |
| Median HD (Q1 to Q3) 2nts 3’ end | 0 (0 to 0) | NR0 (0 to 0) | NR0 (0 to 0) | 0 (0 to 0) | 0 (0 to 0) | NR0 (0 to 0) | NR0 (0 to 0) | 0 (0 to 0) | 0 (0 to 0) | NR0 (0 to 0) | NR0 (0 to 0) | 0 (0 to 0) |
| Percent Qualified Definition #1 | 88.61% | g94.22% | | 99.68% | 95.62% | h93.41% | | 99.66% | 97.25% | i94.75% | | 99.97% |
| Percent Qualified Definition #2 | 87.93% | NR94.22% | | 99.36% | 94.80% | NR93.41% | | 98.95% | 95.28% | NR94.75% | | 96.81% |
| Percent Qualified Definition #3 | 0.85% | 0% | 74.29% | 78.89% | 1.26% | 0.03% | 71.53% | 67.71% | 64.17% | 0.21% | 78.86% | 69.67% |
| NR - Value not relevant for analysis | | | | | | | | | | | | |
(h)
IPDA-BC, env
| Subtype (Country Code) | C (IN) | | | | C (ZA) | | | | B (US) | | | |
| --- | --- | --- | --- | --- | --- | --- | --- | --- | --- | --- | --- | --- |
| Sequence Name | forward-B&C | \*hypermutated probe | intact probe | \*reverse | forward-B&C | \*hypermutated probe | intact probe | \*reverse | forward-B&C | \*hypermutated probe | intact probe | \*reverse |
| Target Sequence 5’-3’ | AGTGGTGSAGAGAGAAAAAAGAGC | CCTTAGGTTCTTAGGAGC | CCTTGGGTTCTTGGGAGC | GCTGACGGTACAGGCCAGAC | AGTGGTGSAGAGAGAAAAAAGAGC | CCTTAGGTTCTTAGGAGC | CCTTGGGTTCTTGGGAGC | GCTGACGGTACAGGCCAGAC | AGTGGTGSAGAGAGAAAAAAGAGC | CCTTAGGTTCTTAGGAGC | CCTTGGGTTCTTGGGAGC | GCTGACGGTACAGGCCAGAC |
| Target Coordinates (HXB2) | 7736-7759 | 7781-7798 | 7781-7798 | 7832-7851 | 7736-7759 | 7781-7798 | 7781-7798 | 7832-7851 | 7736-7759 | 7781-7798 | 7781-7798 | 7832-7851 |
| Sample Size (Genomes) | 1176 | 1229 | | 1246 | 10049 | 10069 | | 10031 | 58806 | 56654 | | 55878 |
| Median HD (Q1 to Q3) Full Length | 0 (0 to 1) | 2 (2 to 3) | 0 (0 to 1) | 0 (0 to 0) | 0 (0 to 1) | 2 (2 to 2) | 0 (0 to 1) | 0 (0 to 1) | 0 (0 to 1) | 2 (2 to 2) | 0 (0 to 1) | 0 (0 to 1) |
| Median HD (Q1 to Q3) 5' Half | 0 (0 to 1) | 1 (1 to 1) | 0 (0 to 0) | 0 (0 to 0) | 0 (0 to 0) | 1 (1 to 1) | 0 (0 to 0) | 0 (0 to 1) | 0 (0 to 0) | 1 (1 to 1) | 0 (0 to 0) | 0 (0 to 1) |
| Median HD (Q1 to Q3) 3' Half | 0 (0 to 1) | 1 (1 to 1) | 0 (0 to 0) | 0 (0 to 0) | 0 (0 to 1) | 1 (1 to 1) | 0 (0 to 0) | 0 (0 to 0) | 0 (0 to 0) | 1 (1 to 1) | 0 (0 to 0) | 0 (0 to 0) |
| Median HD (Q1 to Q3) 2nts 3’ end | 0 (0 to 0) | NR0 (0 to 0) | NR0 (0 to 0) | 0 (0 to 0) | 0 (0 to 0) | NR0 (0 to 0) | NR0 (0 to 0) | 0 (0 to 0) | 0 (0 to 0) | NR0 (0 to 0) | NR0 (0 to 0) | 0 (0 to 0) |
| Percent Qualified Definition #1 | 89.71% | j90.64% | | 99.68% | 95.60% | k91.91% | | 99.66% | 97.25% | l93.43% | | 99.97% |
| Percent Qualified Definition #2 | 89.03% | NR90.64% | | 99.36% | 94.78% | NR91.91% | | 98.95% | 95.28% | NR93.43% | | 96.81% |
| Percent Qualified Definition #3 | 52.98% | 0% | 63.14% | 78.89% | 63.34% | 0.03% | 68.05% | 67.71% | 64.18% | 0.21% | 72.99% | 69.67% |
| NR – Value not relevant for analysis – Analysis identical to IPDA Original | | | | | | | | | | | | |

## Slide 14
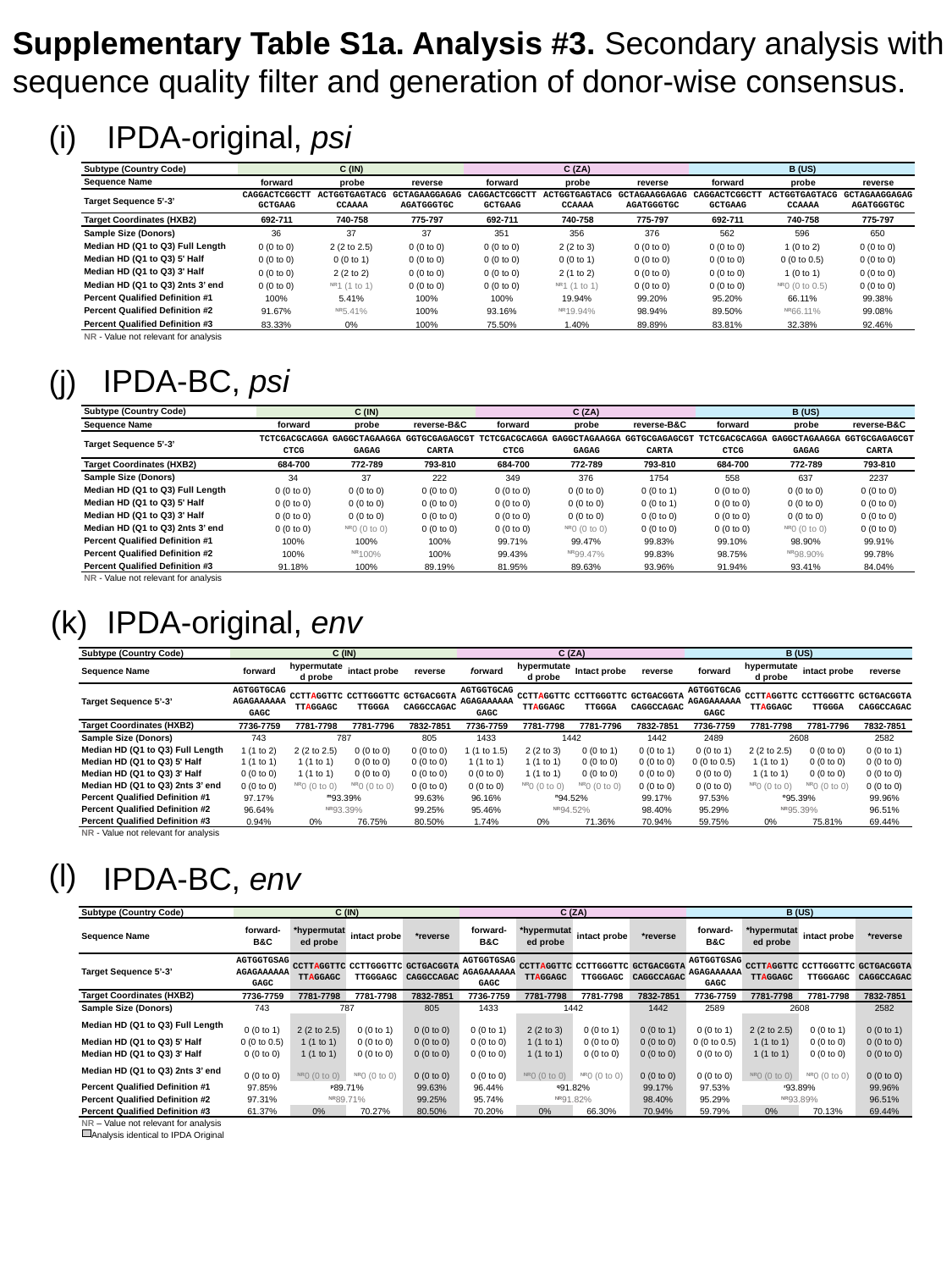

Supplementary Table S1a. Analysis #3. Secondary analysis with sequence quality filter and generation of donor-wise consensus.
IPDA-original, psi
(i)
| Subtype (Country Code) | C (IN) | | | C (ZA) | | | B (US) | | |
| --- | --- | --- | --- | --- | --- | --- | --- | --- | --- |
| Sequence Name | forward | probe | reverse | forward | probe | reverse | forward | probe | reverse |
| Target Sequence 5’-3’ | CAGGACTCGGCTTGCTGAAG | ACTGGTGAGTACGCCAAAA | GCTAGAAGGAGAGAGATGGGTGC | CAGGACTCGGCTTGCTGAAG | ACTGGTGAGTACGCCAAAA | GCTAGAAGGAGAGAGATGGGTGC | CAGGACTCGGCTTGCTGAAG | ACTGGTGAGTACGCCAAAA | GCTAGAAGGAGAGAGATGGGTGC |
| Target Coordinates (HXB2) | 692-711 | 740-758 | 775-797 | 692-711 | 740-758 | 775-797 | 692-711 | 740-758 | 775-797 |
| Sample Size (Donors) | 36 | 37 | 37 | 351 | 356 | 376 | 562 | 596 | 650 |
| Median HD (Q1 to Q3) Full Length | 0 (0 to 0) | 2 (2 to 2.5) | 0 (0 to 0) | 0 (0 to 0) | 2 (2 to 3) | 0 (0 to 0) | 0 (0 to 0) | 1 (0 to 2) | 0 (0 to 0) |
| Median HD (Q1 to Q3) 5' Half | 0 (0 to 0) | 0 (0 to 1) | 0 (0 to 0) | 0 (0 to 0) | 0 (0 to 1) | 0 (0 to 0) | 0 (0 to 0) | 0 (0 to 0.5) | 0 (0 to 0) |
| Median HD (Q1 to Q3) 3' Half | 0 (0 to 0) | 2 (2 to 2) | 0 (0 to 0) | 0 (0 to 0) | 2 (1 to 2) | 0 (0 to 0) | 0 (0 to 0) | 1 (0 to 1) | 0 (0 to 0) |
| Median HD (Q1 to Q3) 2nts 3’ end | 0 (0 to 0) | NR1 (1 to 1) | 0 (0 to 0) | 0 (0 to 0) | NR1 (1 to 1) | 0 (0 to 0) | 0 (0 to 0) | NR0 (0 to 0.5) | 0 (0 to 0) |
| Percent Qualified Definition #1 | 100% | 5.41% | 100% | 100% | 19.94% | 99.20% | 95.20% | 66.11% | 99.38% |
| Percent Qualified Definition #2 | 91.67% | NR5.41% | 100% | 93.16% | NR19.94% | 98.94% | 89.50% | NR66.11% | 99.08% |
| Percent Qualified Definition #3 | 83.33% | 0% | 100% | 75.50% | 1.40% | 89.89% | 83.81% | 32.38% | 92.46% |
| NR - Value not relevant for analysis | | | | | | | | | |
IPDA-BC, psi
(j)
| Subtype (Country Code) | C (IN) | | | C (ZA) | | | B (US) | | |
| --- | --- | --- | --- | --- | --- | --- | --- | --- | --- |
| Sequence Name | forward | probe | reverse-B&C | forward | probe | reverse-B&C | forward | probe | reverse-B&C |
| Target Sequence 5’-3’ | TCTCGACGCAGGACTCG | GAGGCTAGAAGGAGAGAG | GGTGCGAGAGCGTCARTA | TCTCGACGCAGGACTCG | GAGGCTAGAAGGAGAGAG | GGTGCGAGAGCGTCARTA | TCTCGACGCAGGACTCG | GAGGCTAGAAGGAGAGAG | GGTGCGAGAGCGTCARTA |
| Target Coordinates (HXB2) | 684-700 | 772-789 | 793-810 | 684-700 | 772-789 | 793-810 | 684-700 | 772-789 | 793-810 |
| Sample Size (Donors) | 34 | 37 | 222 | 349 | 376 | 1754 | 558 | 637 | 2237 |
| Median HD (Q1 to Q3) Full Length | 0 (0 to 0) | 0 (0 to 0) | 0 (0 to 0) | 0 (0 to 0) | 0 (0 to 0) | 0 (0 to 1) | 0 (0 to 0) | 0 (0 to 0) | 0 (0 to 0) |
| Median HD (Q1 to Q3) 5' Half | 0 (0 to 0) | 0 (0 to 0) | 0 (0 to 0) | 0 (0 to 0) | 0 (0 to 0) | 0 (0 to 1) | 0 (0 to 0) | 0 (0 to 0) | 0 (0 to 0) |
| Median HD (Q1 to Q3) 3' Half | 0 (0 to 0) | 0 (0 to 0) | 0 (0 to 0) | 0 (0 to 0) | 0 (0 to 0) | 0 (0 to 0) | 0 (0 to 0) | 0 (0 to 0) | 0 (0 to 0) |
| Median HD (Q1 to Q3) 2nts 3’ end | 0 (0 to 0) | NR0 (0 to 0) | 0 (0 to 0) | 0 (0 to 0) | NR0 (0 to 0) | 0 (0 to 0) | 0 (0 to 0) | NR0 (0 to 0) | 0 (0 to 0) |
| Percent Qualified Definition #1 | 100% | 100% | 100% | 99.71% | 99.47% | 99.83% | 99.10% | 98.90% | 99.91% |
| Percent Qualified Definition #2 | 100% | NR100% | 100% | 99.43% | NR99.47% | 99.83% | 98.75% | NR98.90% | 99.78% |
| Percent Qualified Definition #3 | 91.18% | 100% | 89.19% | 81.95% | 89.63% | 93.96% | 91.94% | 93.41% | 84.04% |
| NR - Value not relevant for analysis | | | | | | | | | |
(k)
IPDA-original, env
| Subtype (Country Code) | C (IN) | | | | C (ZA) | | | | B (US) | | | |
| --- | --- | --- | --- | --- | --- | --- | --- | --- | --- | --- | --- | --- |
| Sequence Name | forward | hypermutated probe | intact probe | reverse | forward | hypermutated probe | Intact probe | reverse | forward | hypermutated probe | intact probe | reverse |
| Target Sequence 5’-3’ | AGTGGTGCAGAGAGAAAAAAGAGC | CCTTAGGTTCTTAGGAGC | CCTTGGGTTCTTGGGA | GCTGACGGTACAGGCCAGAC | AGTGGTGCAGAGAGAAAAAAGAGC | CCTTAGGTTCTTAGGAGC | CCTTGGGTTCTTGGGA | GCTGACGGTACAGGCCAGAC | AGTGGTGCAGAGAGAAAAAAGAGC | CCTTAGGTTCTTAGGAGC | CCTTGGGTTCTTGGGA | GCTGACGGTACAGGCCAGAC |
| Target Coordinates (HXB2) | 7736-7759 | 7781-7798 | 7781-7796 | 7832-7851 | 7736-7759 | 7781-7798 | 7781-7796 | 7832-7851 | 7736-7759 | 7781-7798 | 7781-7796 | 7832-7851 |
| Sample Size (Donors) | 743 | 787 | | 805 | 1433 | 1442 | | 1442 | 2489 | 2608 | | 2582 |
| Median HD (Q1 to Q3) Full Length | 1 (1 to 2) | 2 (2 to 2.5) | 0 (0 to 0) | 0 (0 to 0) | 1 (1 to 1.5) | 2 (2 to 3) | 0 (0 to 1) | 0 (0 to 1) | 0 (0 to 1) | 2 (2 to 2.5) | 0 (0 to 0) | 0 (0 to 1) |
| Median HD (Q1 to Q3) 5' Half | 1 (1 to 1) | 1 (1 to 1) | 0 (0 to 0) | 0 (0 to 0) | 1 (1 to 1) | 1 (1 to 1) | 0 (0 to 0) | 0 (0 to 0) | 0 (0 to 0.5) | 1 (1 to 1) | 0 (0 to 0) | 0 (0 to 0) |
| Median HD (Q1 to Q3) 3' Half | 0 (0 to 0) | 1 (1 to 1) | 0 (0 to 0) | 0 (0 to 0) | 0 (0 to 0) | 1 (1 to 1) | 0 (0 to 0) | 0 (0 to 0) | 0 (0 to 0) | 1 (1 to 1) | 0 (0 to 0) | 0 (0 to 0) |
| Median HD (Q1 to Q3) 2nts 3’ end | 0 (0 to 0) | NR0 (0 to 0) | NR0 (0 to 0) | 0 (0 to 0) | 0 (0 to 0) | NR0 (0 to 0) | NR0 (0 to 0) | 0 (0 to 0) | 0 (0 to 0) | NR0 (0 to 0) | NR0 (0 to 0) | 0 (0 to 0) |
| Percent Qualified Definition #1 | 97.17% | m93.39% | | 99.63% | 96.16% | n94.52% | | 99.17% | 97.53% | o95.39% | | 99.96% |
| Percent Qualified Definition #2 | 96.64% | NR93.39% | | 99.25% | 95.46% | NR94.52% | | 98.40% | 95.29% | NR95.39% | | 96.51% |
| Percent Qualified Definition #3 | 0.94% | 0% | 76.75% | 80.50% | 1.74% | 0% | 71.36% | 70.94% | 59.75% | 0% | 75.81% | 69.44% |
| NR - Value not relevant for analysis | | | | | | | | | | | | |
(l)
IPDA-BC, env
| Subtype (Country Code) | C (IN) | | | | C (ZA) | | | | B (US) | | | |
| --- | --- | --- | --- | --- | --- | --- | --- | --- | --- | --- | --- | --- |
| Sequence Name | forward-B&C | \*hypermutated probe | intact probe | \*reverse | forward-B&C | \*hypermutated probe | intact probe | \*reverse | forward-B&C | \*hypermutated probe | intact probe | \*reverse |
| Target Sequence 5’-3’ | AGTGGTGSAGAGAGAAAAAAGAGC | CCTTAGGTTCTTAGGAGC | CCTTGGGTTCTTGGGAGC | GCTGACGGTACAGGCCAGAC | AGTGGTGSAGAGAGAAAAAAGAGC | CCTTAGGTTCTTAGGAGC | CCTTGGGTTCTTGGGAGC | GCTGACGGTACAGGCCAGAC | AGTGGTGSAGAGAGAAAAAAGAGC | CCTTAGGTTCTTAGGAGC | CCTTGGGTTCTTGGGAGC | GCTGACGGTACAGGCCAGAC |
| Target Coordinates (HXB2) | 7736-7759 | 7781-7798 | 7781-7798 | 7832-7851 | 7736-7759 | 7781-7798 | 7781-7798 | 7832-7851 | 7736-7759 | 7781-7798 | 7781-7798 | 7832-7851 |
| Sample Size (Donors) | 743 | 787 | | 805 | 1433 | 1442 | | 1442 | 2589 | 2608 | | 2582 |
| Median HD (Q1 to Q3) Full Length | 0 (0 to 1) | 2 (2 to 2.5) | 0 (0 to 1) | 0 (0 to 0) | 0 (0 to 1) | 2 (2 to 3) | 0 (0 to 1) | 0 (0 to 1) | 0 (0 to 1) | 2 (2 to 2.5) | 0 (0 to 1) | 0 (0 to 1) |
| Median HD (Q1 to Q3) 5' Half | 0 (0 to 0.5) | 1 (1 to 1) | 0 (0 to 0) | 0 (0 to 0) | 0 (0 to 0) | 1 (1 to 1) | 0 (0 to 0) | 0 (0 to 0) | 0 (0 to 0.5) | 1 (1 to 1) | 0 (0 to 0) | 0 (0 to 0) |
| Median HD (Q1 to Q3) 3' Half | 0 (0 to 0) | 1 (1 to 1) | 0 (0 to 0) | 0 (0 to 0) | 0 (0 to 0) | 1 (1 to 1) | 0 (0 to 0) | 0 (0 to 0) | 0 (0 to 0) | 1 (1 to 1) | 0 (0 to 0) | 0 (0 to 0) |
| Median HD (Q1 to Q3) 2nts 3’ end | 0 (0 to 0) | NR0 (0 to 0) | NR0 (0 to 0) | 0 (0 to 0) | 0 (0 to 0) | NR0 (0 to 0) | NR0 (0 to 0) | 0 (0 to 0) | 0 (0 to 0) | NR0 (0 to 0) | NR0 (0 to 0) | 0 (0 to 0) |
| Percent Qualified Definition #1 | 97.85% | p89.71% | | 99.63% | 96.44% | q91.82% | | 99.17% | 97.53% | r93.89% | | 99.96% |
| Percent Qualified Definition #2 | 97.31% | NR89.71% | | 99.25% | 95.74% | NR91.82% | | 98.40% | 95.29% | NR93.89% | | 96.51% |
| Percent Qualified Definition #3 | 61.37% | 0% | 70.27% | 80.50% | 70.20% | 0% | 66.30% | 70.94% | 59.79% | 0% | 70.13% | 69.44% |
| NR – Value not relevant for analysis – Analysis identical to IPDA Original | | | | | | | | | | | | |

## Slide 15
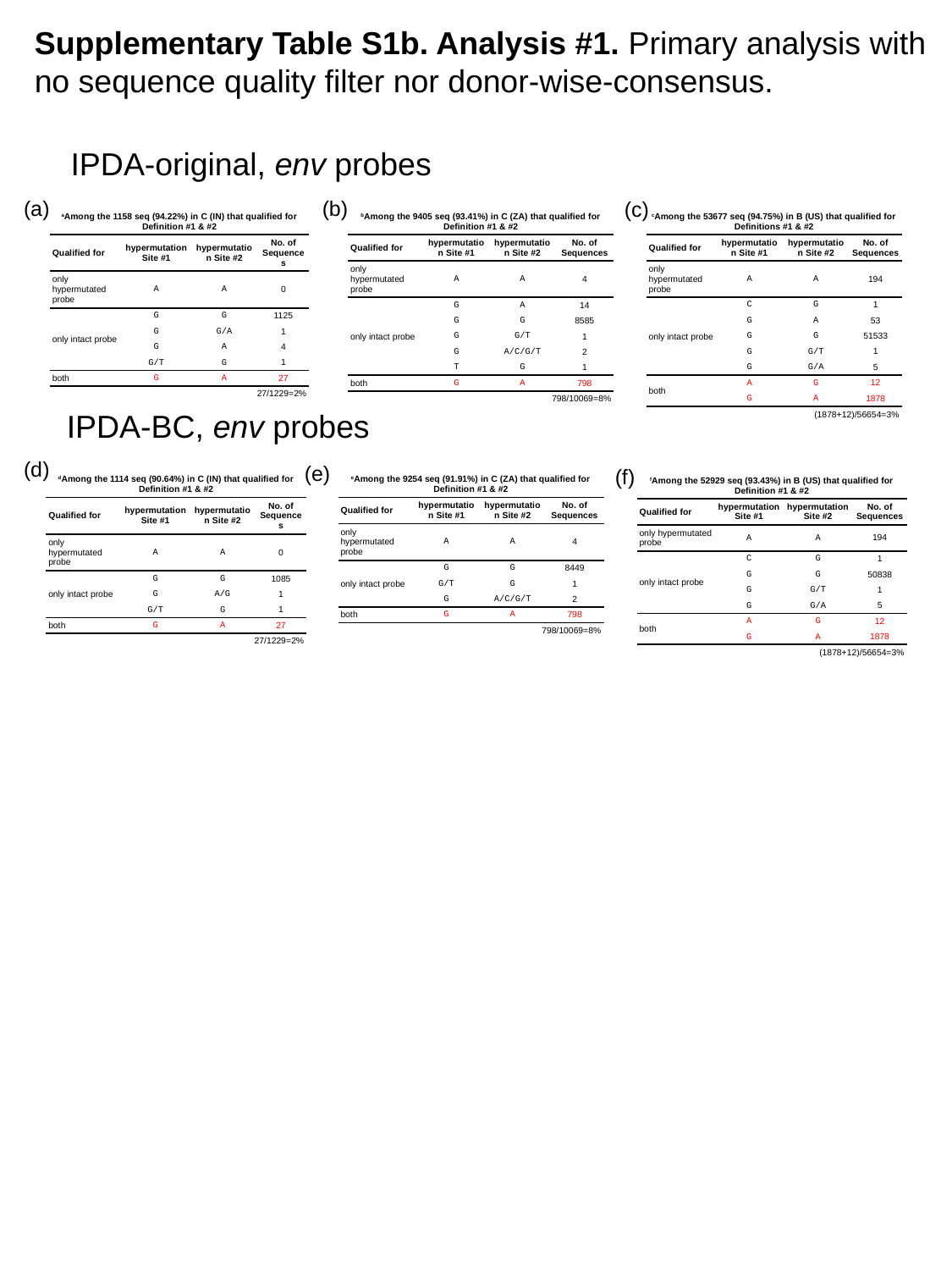

Supplementary Table S1b. Analysis #1. Primary analysis with no sequence quality filter nor donor-wise-consensus.
IPDA-original, env probes
(a)
(b)
(c)
| aAmong the 1158 seq (94.22%) in C (IN) that qualified for Definition #1 & #2 | | | |
| --- | --- | --- | --- |
| Qualified for | hypermutation Site #1 | hypermutation Site #2 | No. of Sequences |
| only hypermutated probe | A | A | 0 |
| only intact probe | G | G | 1125 |
| | G | G/A | 1 |
| | G | A | 4 |
| | G/T | G | 1 |
| both | G | A | 27 |
| 27/1229=2% | | | |
| bAmong the 9405 seq (93.41%) in C (ZA) that qualified for Definition #1 & #2 | | | |
| --- | --- | --- | --- |
| Qualified for | hypermutation Site #1 | hypermutation Site #2 | No. of Sequences |
| only hypermutated probe | A | A | 4 |
| only intact probe | G | A | 14 |
| | G | G | 8585 |
| | G | G/T | 1 |
| | G | A/C/G/T | 2 |
| | T | G | 1 |
| both | G | A | 798 |
| 798/10069=8% | | | |
| cAmong the 53677 seq (94.75%) in B (US) that qualified for Definitions #1 & #2 | | | |
| --- | --- | --- | --- |
| Qualified for | hypermutation Site #1 | hypermutation Site #2 | No. of Sequences |
| only hypermutated probe | A | A | 194 |
| only intact probe | C | G | 1 |
| | G | A | 53 |
| | G | G | 51533 |
| | G | G/T | 1 |
| | G | G/A | 5 |
| both | A | G | 12 |
| | G | A | 1878 |
| (1878+12)/56654=3% | | | |
IPDA-BC, env probes
(d)
(e)
(f)
| dAmong the 1114 seq (90.64%) in C (IN) that qualified for Definition #1 & #2 | | | |
| --- | --- | --- | --- |
| Qualified for | hypermutation Site #1 | hypermutation Site #2 | No. of Sequences |
| only hypermutated probe | A | A | 0 |
| only intact probe | G | G | 1085 |
| | G | A/G | 1 |
| | G/T | G | 1 |
| both | G | A | 27 |
| 27/1229=2% | | | |
| eAmong the 9254 seq (91.91%) in C (ZA) that qualified for Definition #1 & #2 | | | |
| --- | --- | --- | --- |
| Qualified for | hypermutation Site #1 | hypermutation Site #2 | No. of Sequences |
| only hypermutated probe | A | A | 4 |
| only intact probe | G | G | 8449 |
| | G/T | G | 1 |
| | G | A/C/G/T | 2 |
| both | G | A | 798 |
| 798/10069=8% | | | |
| fAmong the 52929 seq (93.43%) in B (US) that qualified for Definition #1 & #2 | | | |
| --- | --- | --- | --- |
| Qualified for | hypermutation Site #1 | hypermutation Site #2 | No. of Sequences |
| only hypermutated probe | A | A | 194 |
| only intact probe | C | G | 1 |
| | G | G | 50838 |
| | G | G/T | 1 |
| | G | G/A | 5 |
| both | A | G | 12 |
| | G | A | 1878 |
| (1878+12)/56654=3% | | | |

## Slide 16
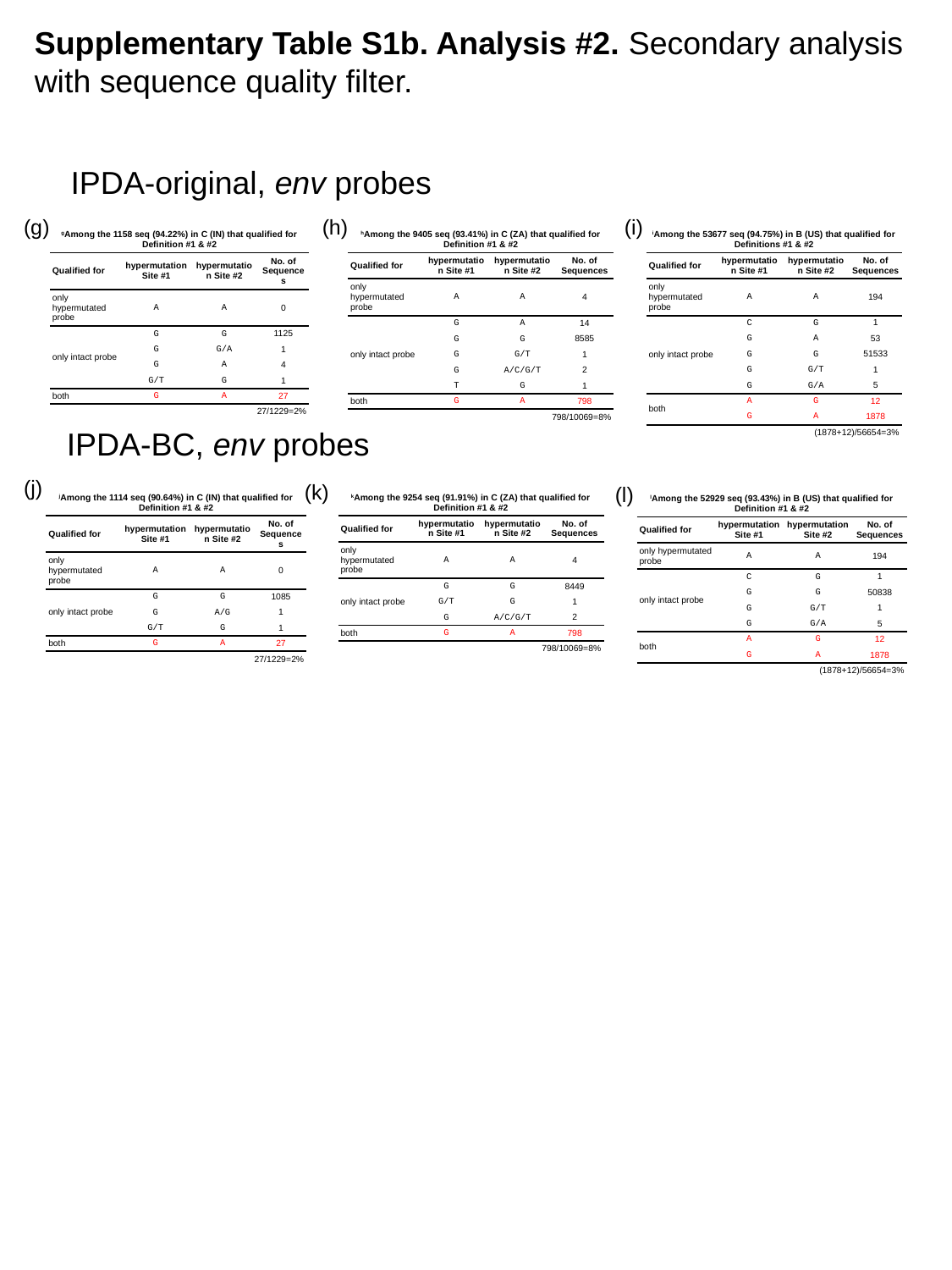

Supplementary Table S1b. Analysis #2. Secondary analysis with sequence quality filter.
IPDA-original, env probes
(g)
(h)
(i)
| gAmong the 1158 seq (94.22%) in C (IN) that qualified for Definition #1 & #2 | | | |
| --- | --- | --- | --- |
| Qualified for | hypermutation Site #1 | hypermutation Site #2 | No. of Sequences |
| only hypermutated probe | A | A | 0 |
| only intact probe | G | G | 1125 |
| | G | G/A | 1 |
| | G | A | 4 |
| | G/T | G | 1 |
| both | G | A | 27 |
| 27/1229=2% | | | |
| hAmong the 9405 seq (93.41%) in C (ZA) that qualified for Definition #1 & #2 | | | |
| --- | --- | --- | --- |
| Qualified for | hypermutation Site #1 | hypermutation Site #2 | No. of Sequences |
| only hypermutated probe | A | A | 4 |
| only intact probe | G | A | 14 |
| | G | G | 8585 |
| | G | G/T | 1 |
| | G | A/C/G/T | 2 |
| | T | G | 1 |
| both | G | A | 798 |
| 798/10069=8% | | | |
| iAmong the 53677 seq (94.75%) in B (US) that qualified for Definitions #1 & #2 | | | |
| --- | --- | --- | --- |
| Qualified for | hypermutation Site #1 | hypermutation Site #2 | No. of Sequences |
| only hypermutated probe | A | A | 194 |
| only intact probe | C | G | 1 |
| | G | A | 53 |
| | G | G | 51533 |
| | G | G/T | 1 |
| | G | G/A | 5 |
| both | A | G | 12 |
| | G | A | 1878 |
| (1878+12)/56654=3% | | | |
IPDA-BC, env probes
(j)
(k)
(l)
| jAmong the 1114 seq (90.64%) in C (IN) that qualified for Definition #1 & #2 | | | |
| --- | --- | --- | --- |
| Qualified for | hypermutation Site #1 | hypermutation Site #2 | No. of Sequences |
| only hypermutated probe | A | A | 0 |
| only intact probe | G | G | 1085 |
| | G | A/G | 1 |
| | G/T | G | 1 |
| both | G | A | 27 |
| 27/1229=2% | | | |
| kAmong the 9254 seq (91.91%) in C (ZA) that qualified for Definition #1 & #2 | | | |
| --- | --- | --- | --- |
| Qualified for | hypermutation Site #1 | hypermutation Site #2 | No. of Sequences |
| only hypermutated probe | A | A | 4 |
| only intact probe | G | G | 8449 |
| | G/T | G | 1 |
| | G | A/C/G/T | 2 |
| both | G | A | 798 |
| 798/10069=8% | | | |
| lAmong the 52929 seq (93.43%) in B (US) that qualified for Definition #1 & #2 | | | |
| --- | --- | --- | --- |
| Qualified for | hypermutation Site #1 | hypermutation Site #2 | No. of Sequences |
| only hypermutated probe | A | A | 194 |
| only intact probe | C | G | 1 |
| | G | G | 50838 |
| | G | G/T | 1 |
| | G | G/A | 5 |
| both | A | G | 12 |
| | G | A | 1878 |
| (1878+12)/56654=3% | | | |

## Slide 17
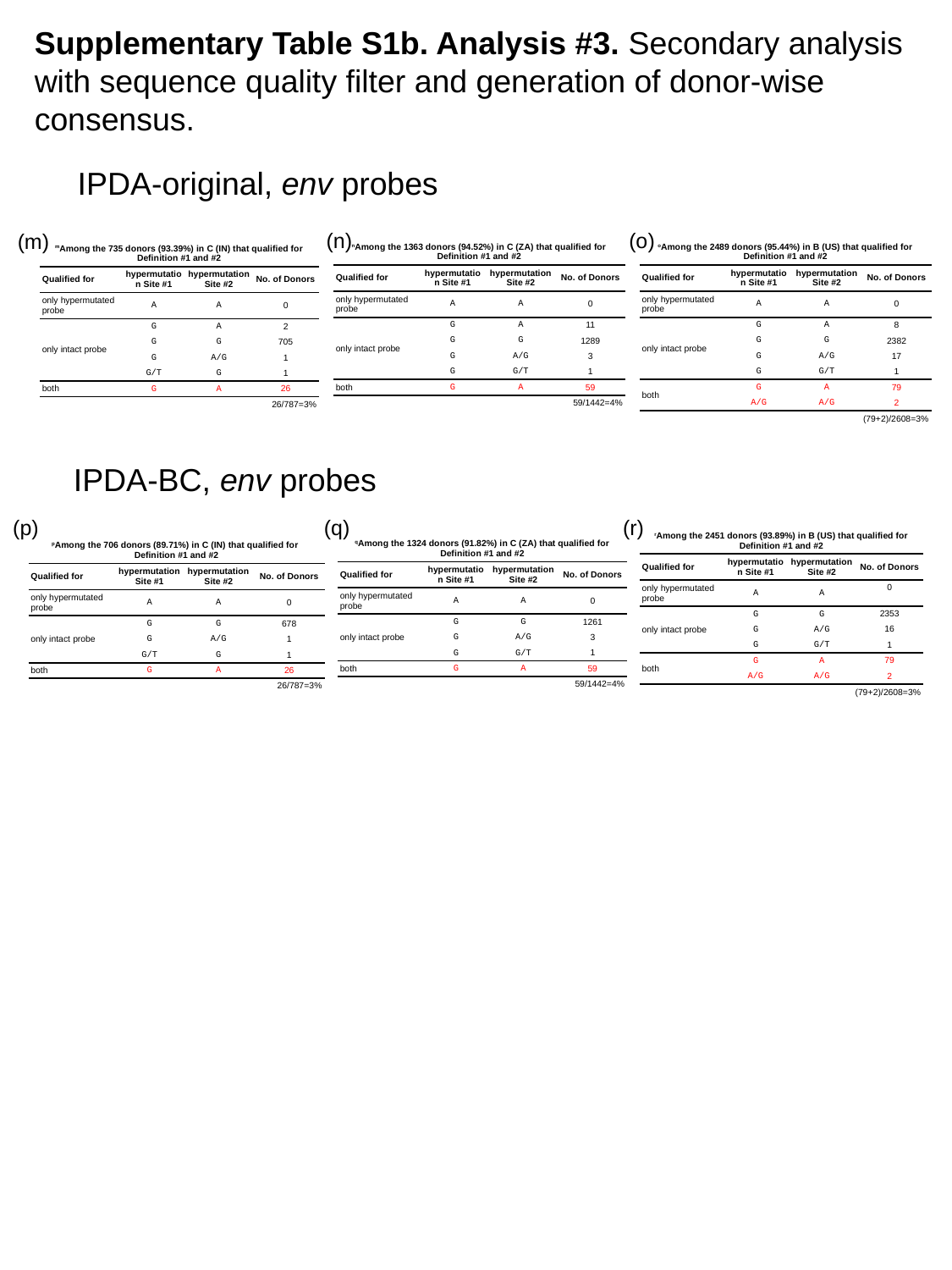

Supplementary Table S1b. Analysis #3. Secondary analysis with sequence quality filter and generation of donor-wise consensus.
IPDA-original, env probes
(n)
(o)
(m)
| oAmong the 2489 donors (95.44%) in B (US) that qualified for Definition #1 and #2 | | | |
| --- | --- | --- | --- |
| Qualified for | hypermutation Site #1 | hypermutation Site #2 | No. of Donors |
| only hypermutated probe | A | A | 0 |
| only intact probe | G | A | 8 |
| | G | G | 2382 |
| | G | A/G | 17 |
| | G | G/T | 1 |
| both | G | A | 79 |
| | A/G | A/G | 2 |
| (79+2)/2608=3% | | | |
| nAmong the 1363 donors (94.52%) in C (ZA) that qualified for Definition #1 and #2 | | | |
| --- | --- | --- | --- |
| Qualified for | hypermutation Site #1 | hypermutation Site #2 | No. of Donors |
| only hypermutated probe | A | A | 0 |
| only intact probe | G | A | 11 |
| | G | G | 1289 |
| | G | A/G | 3 |
| | G | G/T | 1 |
| both | G | A | 59 |
| 59/1442=4% | | | |
| mAmong the 735 donors (93.39%) in C (IN) that qualified for Definition #1 and #2 | | | |
| --- | --- | --- | --- |
| Qualified for | hypermutation Site #1 | hypermutation Site #2 | No. of Donors |
| only hypermutated probe | A | A | 0 |
| only intact probe | G | A | 2 |
| | G | G | 705 |
| | G | A/G | 1 |
| | G/T | G | 1 |
| both | G | A | 26 |
| 26/787=3% | | | |
IPDA-BC, env probes
(p)
(q)
(r)
| rAmong the 2451 donors (93.89%) in B (US) that qualified for Definition #1 and #2 | | | |
| --- | --- | --- | --- |
| Qualified for | hypermutation Site #1 | hypermutation Site #2 | No. of Donors |
| only hypermutated probe | A | A | 0 |
| only intact probe | G | G | 2353 |
| | G | A/G | 16 |
| | G | G/T | 1 |
| both | G | A | 79 |
| | A/G | A/G | 2 |
| (79+2)/2608=3% | | | |
| qAmong the 1324 donors (91.82%) in C (ZA) that qualified for Definition #1 and #2 | | | |
| --- | --- | --- | --- |
| Qualified for | hypermutation Site #1 | hypermutation Site #2 | No. of Donors |
| only hypermutated probe | A | A | 0 |
| only intact probe | G | G | 1261 |
| | G | A/G | 3 |
| | G | G/T | 1 |
| both | G | A | 59 |
| 59/1442=4% | | | |
| pAmong the 706 donors (89.71%) in C (IN) that qualified for Definition #1 and #2 | | | |
| --- | --- | --- | --- |
| Qualified for | hypermutation Site #1 | hypermutation Site #2 | No. of Donors |
| only hypermutated probe | A | A | 0 |
| only intact probe | G | G | 678 |
| | G | A/G | 1 |
| | G/T | G | 1 |
| both | G | A | 26 |
| 26/787=3% | | | |

## Slide 18
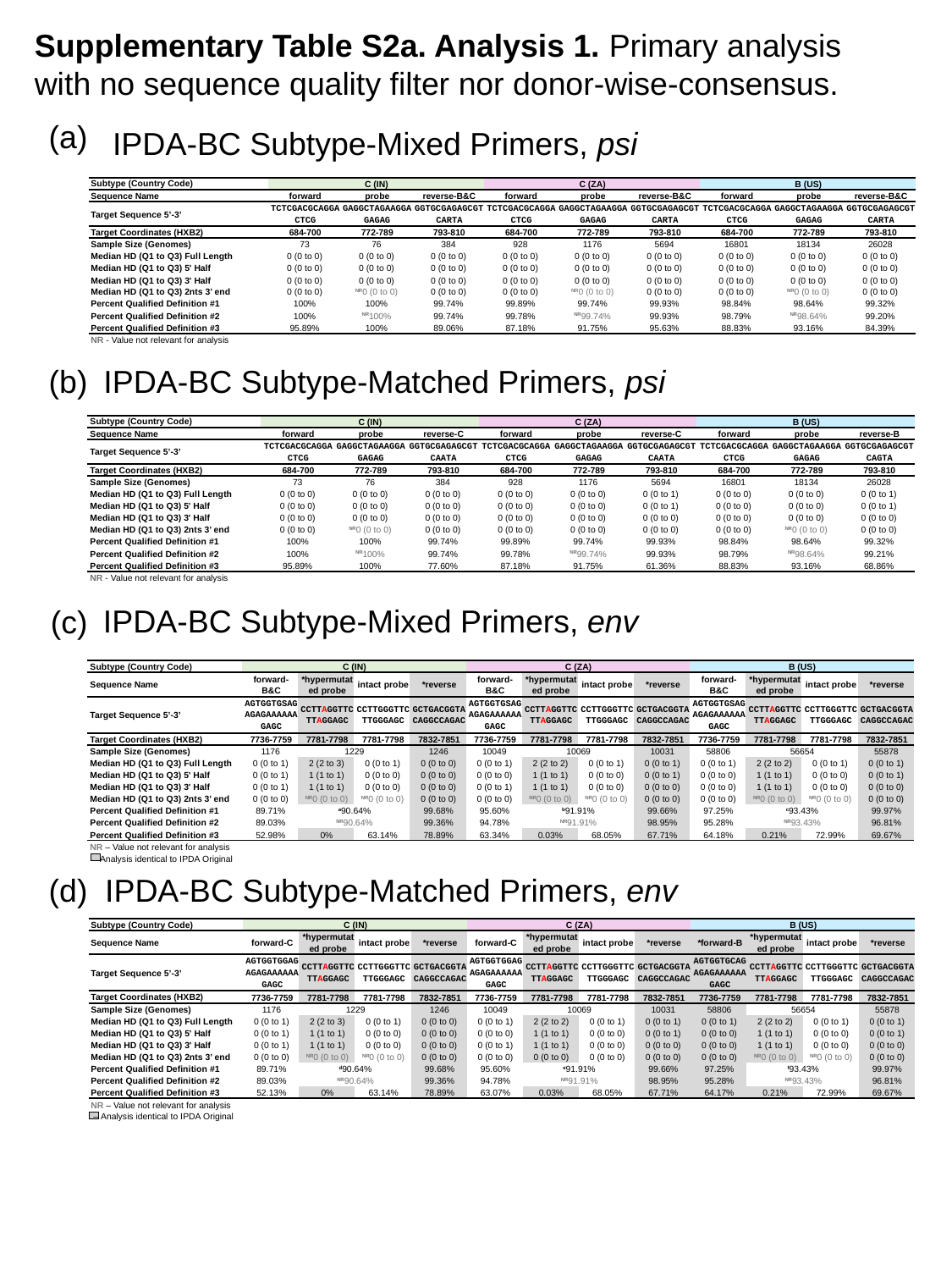

Supplementary Table S2a. Analysis 1. Primary analysis with no sequence quality filter nor donor-wise-consensus.
(a)
IPDA-BC Subtype-Mixed Primers, psi
| Subtype (Country Code) | C (IN) | | | C (ZA) | | | B (US) | | |
| --- | --- | --- | --- | --- | --- | --- | --- | --- | --- |
| Sequence Name | forward | probe | reverse-B&C | forward | probe | reverse-B&C | forward | probe | reverse-B&C |
| Target Sequence 5’-3’ | TCTCGACGCAGGACTCG | GAGGCTAGAAGGAGAGAG | GGTGCGAGAGCGTCARTA | TCTCGACGCAGGACTCG | GAGGCTAGAAGGAGAGAG | GGTGCGAGAGCGTCARTA | TCTCGACGCAGGACTCG | GAGGCTAGAAGGAGAGAG | GGTGCGAGAGCGTCARTA |
| Target Coordinates (HXB2) | 684-700 | 772-789 | 793-810 | 684-700 | 772-789 | 793-810 | 684-700 | 772-789 | 793-810 |
| Sample Size (Genomes) | 73 | 76 | 384 | 928 | 1176 | 5694 | 16801 | 18134 | 26028 |
| Median HD (Q1 to Q3) Full Length | 0 (0 to 0) | 0 (0 to 0) | 0 (0 to 0) | 0 (0 to 0) | 0 (0 to 0) | 0 (0 to 0) | 0 (0 to 0) | 0 (0 to 0) | 0 (0 to 0) |
| Median HD (Q1 to Q3) 5' Half | 0 (0 to 0) | 0 (0 to 0) | 0 (0 to 0) | 0 (0 to 0) | 0 (0 to 0) | 0 (0 to 0) | 0 (0 to 0) | 0 (0 to 0) | 0 (0 to 0) |
| Median HD (Q1 to Q3) 3' Half | 0 (0 to 0) | 0 (0 to 0) | 0 (0 to 0) | 0 (0 to 0) | 0 (0 to 0) | 0 (0 to 0) | 0 (0 to 0) | 0 (0 to 0) | 0 (0 to 0) |
| Median HD (Q1 to Q3) 2nts 3’ end | 0 (0 to 0) | NR0 (0 to 0) | 0 (0 to 0) | 0 (0 to 0) | NR0 (0 to 0) | 0 (0 to 0) | 0 (0 to 0) | NR0 (0 to 0) | 0 (0 to 0) |
| Percent Qualified Definition #1 | 100% | 100% | 99.74% | 99.89% | 99.74% | 99.93% | 98.84% | 98.64% | 99.32% |
| Percent Qualified Definition #2 | 100% | NR100% | 99.74% | 99.78% | NR99.74% | 99.93% | 98.79% | NR98.64% | 99.20% |
| Percent Qualified Definition #3 | 95.89% | 100% | 89.06% | 87.18% | 91.75% | 95.63% | 88.83% | 93.16% | 84.39% |
| NR - Value not relevant for analysis | | | | | | | | | |
IPDA-BC Subtype-Matched Primers, psi
(b)
| Subtype (Country Code) | C (IN) | | | C (ZA) | | | B (US) | | |
| --- | --- | --- | --- | --- | --- | --- | --- | --- | --- |
| Sequence Name | forward | probe | reverse-C | forward | probe | reverse-C | forward | probe | reverse-B |
| Target Sequence 5’-3’ | TCTCGACGCAGGACTCG | GAGGCTAGAAGGAGAGAG | GGTGCGAGAGCGTCAATA | TCTCGACGCAGGACTCG | GAGGCTAGAAGGAGAGAG | GGTGCGAGAGCGTCAATA | TCTCGACGCAGGACTCG | GAGGCTAGAAGGAGAGAG | GGTGCGAGAGCGTCAGTA |
| Target Coordinates (HXB2) | 684-700 | 772-789 | 793-810 | 684-700 | 772-789 | 793-810 | 684-700 | 772-789 | 793-810 |
| Sample Size (Genomes) | 73 | 76 | 384 | 928 | 1176 | 5694 | 16801 | 18134 | 26028 |
| Median HD (Q1 to Q3) Full Length | 0 (0 to 0) | 0 (0 to 0) | 0 (0 to 0) | 0 (0 to 0) | 0 (0 to 0) | 0 (0 to 1) | 0 (0 to 0) | 0 (0 to 0) | 0 (0 to 1) |
| Median HD (Q1 to Q3) 5' Half | 0 (0 to 0) | 0 (0 to 0) | 0 (0 to 0) | 0 (0 to 0) | 0 (0 to 0) | 0 (0 to 1) | 0 (0 to 0) | 0 (0 to 0) | 0 (0 to 1) |
| Median HD (Q1 to Q3) 3' Half | 0 (0 to 0) | 0 (0 to 0) | 0 (0 to 0) | 0 (0 to 0) | 0 (0 to 0) | 0 (0 to 0) | 0 (0 to 0) | 0 (0 to 0) | 0 (0 to 0) |
| Median HD (Q1 to Q3) 2nts 3’ end | 0 (0 to 0) | NR0 (0 to 0) | 0 (0 to 0) | 0 (0 to 0) | 0 (0 to 0) | 0 (0 to 0) | 0 (0 to 0) | NR0 (0 to 0) | 0 (0 to 0) |
| Percent Qualified Definition #1 | 100% | 100% | 99.74% | 99.89% | 99.74% | 99.93% | 98.84% | 98.64% | 99.32% |
| Percent Qualified Definition #2 | 100% | NR100% | 99.74% | 99.78% | NR99.74% | 99.93% | 98.79% | NR98.64% | 99.21% |
| Percent Qualified Definition #3 | 95.89% | 100% | 77.60% | 87.18% | 91.75% | 61.36% | 88.83% | 93.16% | 68.86% |
| NR - Value not relevant for analysis | | | | | | | | | |
IPDA-BC Subtype-Mixed Primers, env
(c)
| Subtype (Country Code) | C (IN) | | | | C (ZA) | | | | B (US) | | | |
| --- | --- | --- | --- | --- | --- | --- | --- | --- | --- | --- | --- | --- |
| Sequence Name | forward-B&C | \*hypermutated probe | intact probe | \*reverse | forward-B&C | \*hypermutated probe | intact probe | \*reverse | forward-B&C | \*hypermutated probe | intact probe | \*reverse |
| Target Sequence 5’-3’ | AGTGGTGSAGAGAGAAAAAAGAGC | CCTTAGGTTCTTAGGAGC | CCTTGGGTTCTTGGGAGC | GCTGACGGTACAGGCCAGAC | AGTGGTGSAGAGAGAAAAAAGAGC | CCTTAGGTTCTTAGGAGC | CCTTGGGTTCTTGGGAGC | GCTGACGGTACAGGCCAGAC | AGTGGTGSAGAGAGAAAAAAGAGC | CCTTAGGTTCTTAGGAGC | CCTTGGGTTCTTGGGAGC | GCTGACGGTACAGGCCAGAC |
| Target Coordinates (HXB2) | 7736-7759 | 7781-7798 | 7781-7798 | 7832-7851 | 7736-7759 | 7781-7798 | 7781-7798 | 7832-7851 | 7736-7759 | 7781-7798 | 7781-7798 | 7832-7851 |
| Sample Size (Genomes) | 1176 | 1229 | | 1246 | 10049 | 10069 | | 10031 | 58806 | 56654 | | 55878 |
| Median HD (Q1 to Q3) Full Length | 0 (0 to 1) | 2 (2 to 3) | 0 (0 to 1) | 0 (0 to 0) | 0 (0 to 1) | 2 (2 to 2) | 0 (0 to 1) | 0 (0 to 1) | 0 (0 to 1) | 2 (2 to 2) | 0 (0 to 1) | 0 (0 to 1) |
| Median HD (Q1 to Q3) 5' Half | 0 (0 to 1) | 1 (1 to 1) | 0 (0 to 0) | 0 (0 to 0) | 0 (0 to 0) | 1 (1 to 1) | 0 (0 to 0) | 0 (0 to 1) | 0 (0 to 0) | 1 (1 to 1) | 0 (0 to 0) | 0 (0 to 1) |
| Median HD (Q1 to Q3) 3' Half | 0 (0 to 1) | 1 (1 to 1) | 0 (0 to 0) | 0 (0 to 0) | 0 (0 to 1) | 1 (1 to 1) | 0 (0 to 0) | 0 (0 to 0) | 0 (0 to 0) | 1 (1 to 1) | 0 (0 to 0) | 0 (0 to 0) |
| Median HD (Q1 to Q3) 2nts 3’ end | 0 (0 to 0) | NR0 (0 to 0) | NR0 (0 to 0) | 0 (0 to 0) | 0 (0 to 0) | NR0 (0 to 0) | NR0 (0 to 0) | 0 (0 to 0) | 0 (0 to 0) | NR0 (0 to 0) | NR0 (0 to 0) | 0 (0 to 0) |
| Percent Qualified Definition #1 | 89.71% | a90.64% | | 99.68% | 95.60% | b91.91% | | 99.66% | 97.25% | c93.43% | | 99.97% |
| Percent Qualified Definition #2 | 89.03% | NR90.64% | | 99.36% | 94.78% | NR91.91% | | 98.95% | 95.28% | NR93.43% | | 96.81% |
| Percent Qualified Definition #3 | 52.98% | 0% | 63.14% | 78.89% | 63.34% | 0.03% | 68.05% | 67.71% | 64.18% | 0.21% | 72.99% | 69.67% |
| NR – Value not relevant for analysis – Analysis identical to IPDA Original | | | | | | | | | | | | |
(d)
IPDA-BC Subtype-Matched Primers, env
| Subtype (Country Code) | C (IN) | | | | C (ZA) | | | | B (US) | | | |
| --- | --- | --- | --- | --- | --- | --- | --- | --- | --- | --- | --- | --- |
| Sequence Name | forward-C | \*hypermutated probe | intact probe | \*reverse | forward-C | \*hypermutated probe | intact probe | \*reverse | \*forward-B | \*hypermutated probe | intact probe | \*reverse |
| Target Sequence 5’-3’ | AGTGGTGGAGAGAGAAAAAAGAGC | CCTTAGGTTCTTAGGAGC | CCTTGGGTTCTTGGGAGC | GCTGACGGTACAGGCCAGAC | AGTGGTGGAGAGAGAAAAAAGAGC | CCTTAGGTTCTTAGGAGC | CCTTGGGTTCTTGGGAGC | GCTGACGGTACAGGCCAGAC | AGTGGTGCAGAGAGAAAAAAGAGC | CCTTAGGTTCTTAGGAGC | CCTTGGGTTCTTGGGAGC | GCTGACGGTACAGGCCAGAC |
| Target Coordinates (HXB2) | 7736-7759 | 7781-7798 | 7781-7798 | 7832-7851 | 7736-7759 | 7781-7798 | 7781-7798 | 7832-7851 | 7736-7759 | 7781-7798 | 7781-7798 | 7832-7851 |
| Sample Size (Genomes) | 1176 | 1229 | | 1246 | 10049 | 10069 | | 10031 | 58806 | 56654 | | 55878 |
| Median HD (Q1 to Q3) Full Length | 0 (0 to 1) | 2 (2 to 3) | 0 (0 to 1) | 0 (0 to 0) | 0 (0 to 1) | 2 (2 to 2) | 0 (0 to 1) | 0 (0 to 1) | 0 (0 to 1) | 2 (2 to 2) | 0 (0 to 1) | 0 (0 to 1) |
| Median HD (Q1 to Q3) 5' Half | 0 (0 to 1) | 1 (1 to 1) | 0 (0 to 0) | 0 (0 to 0) | 0 (0 to 0) | 1 (1 to 1) | 0 (0 to 0) | 0 (0 to 1) | 0 (0 to 0) | 1 (1 to 1) | 0 (0 to 0) | 0 (0 to 1) |
| Median HD (Q1 to Q3) 3' Half | 0 (0 to 1) | 1 (1 to 1) | 0 (0 to 0) | 0 (0 to 0) | 0 (0 to 1) | 1 (1 to 1) | 0 (0 to 0) | 0 (0 to 0) | 0 (0 to 0) | 1 (1 to 1) | 0 (0 to 0) | 0 (0 to 0) |
| Median HD (Q1 to Q3) 2nts 3’ end | 0 (0 to 0) | NR0 (0 to 0) | NR0 (0 to 0) | 0 (0 to 0) | 0 (0 to 0) | 0 (0 to 0) | 0 (0 to 0) | 0 (0 to 0) | 0 (0 to 0) | NR0 (0 to 0) | NR0 (0 to 0) | 0 (0 to 0) |
| Percent Qualified Definition #1 | 89.71% | d90.64% | | 99.68% | 95.60% | e91.91% | | 99.66% | 97.25% | f93.43% | | 99.97% |
| Percent Qualified Definition #2 | 89.03% | NR90.64% | | 99.36% | 94.78% | NR91.91% | | 98.95% | 95.28% | NR93.43% | | 96.81% |
| Percent Qualified Definition #3 | 52.13% | 0% | 63.14% | 78.89% | 63.07% | 0.03% | 68.05% | 67.71% | 64.17% | 0.21% | 72.99% | 69.67% |
| NR – Value not relevant for analysis – Analysis identical to IPDA Original | | | | | | | | | | | | |

## Slide 19
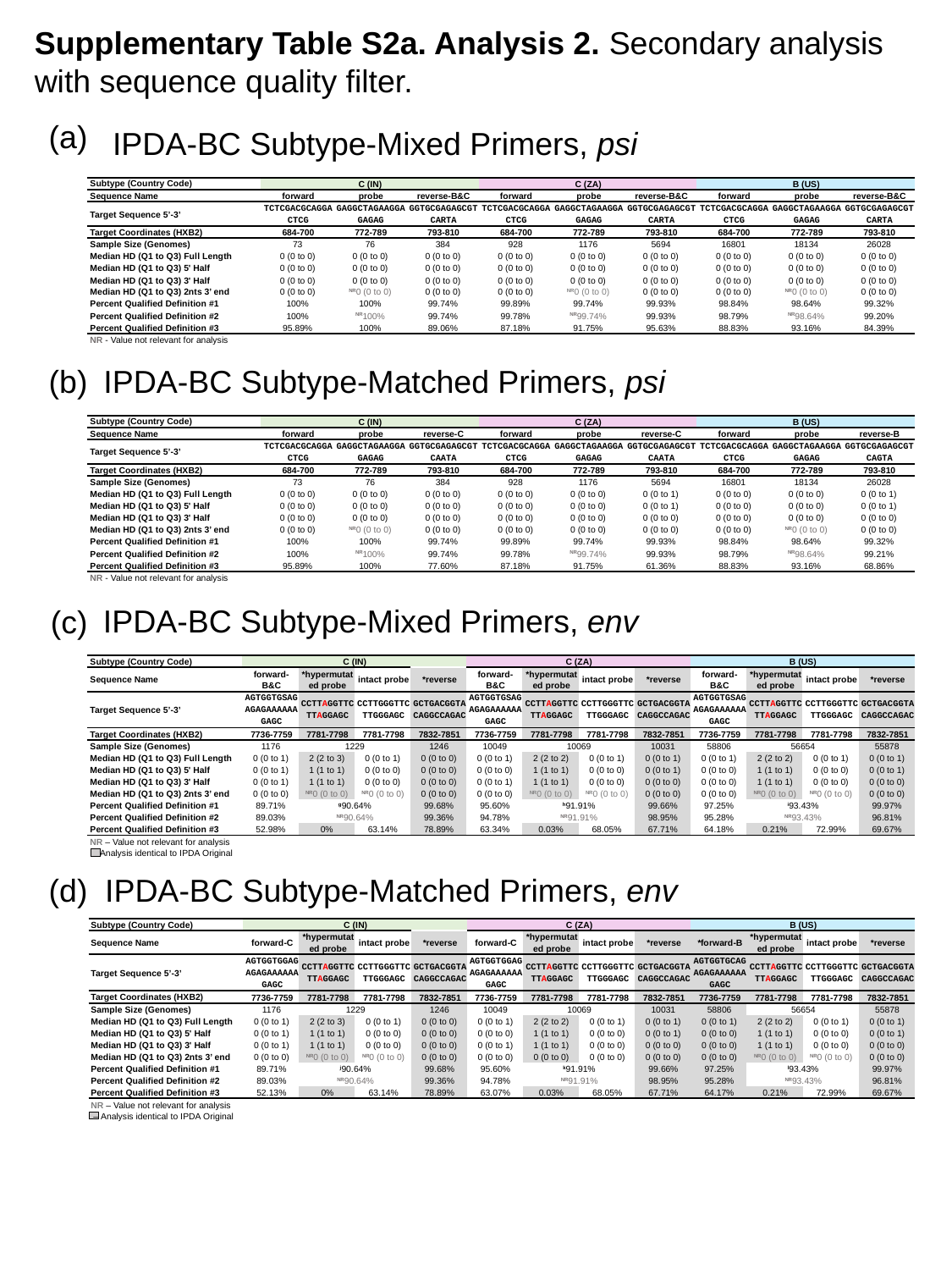

Supplementary Table S2a. Analysis 2. Secondary analysis with sequence quality filter.
(a)
IPDA-BC Subtype-Mixed Primers, psi
| Subtype (Country Code) | C (IN) | | | C (ZA) | | | B (US) | | |
| --- | --- | --- | --- | --- | --- | --- | --- | --- | --- |
| Sequence Name | forward | probe | reverse-B&C | forward | probe | reverse-B&C | forward | probe | reverse-B&C |
| Target Sequence 5’-3’ | TCTCGACGCAGGACTCG | GAGGCTAGAAGGAGAGAG | GGTGCGAGAGCGTCARTA | TCTCGACGCAGGACTCG | GAGGCTAGAAGGAGAGAG | GGTGCGAGAGCGTCARTA | TCTCGACGCAGGACTCG | GAGGCTAGAAGGAGAGAG | GGTGCGAGAGCGTCARTA |
| Target Coordinates (HXB2) | 684-700 | 772-789 | 793-810 | 684-700 | 772-789 | 793-810 | 684-700 | 772-789 | 793-810 |
| Sample Size (Genomes) | 73 | 76 | 384 | 928 | 1176 | 5694 | 16801 | 18134 | 26028 |
| Median HD (Q1 to Q3) Full Length | 0 (0 to 0) | 0 (0 to 0) | 0 (0 to 0) | 0 (0 to 0) | 0 (0 to 0) | 0 (0 to 0) | 0 (0 to 0) | 0 (0 to 0) | 0 (0 to 0) |
| Median HD (Q1 to Q3) 5' Half | 0 (0 to 0) | 0 (0 to 0) | 0 (0 to 0) | 0 (0 to 0) | 0 (0 to 0) | 0 (0 to 0) | 0 (0 to 0) | 0 (0 to 0) | 0 (0 to 0) |
| Median HD (Q1 to Q3) 3' Half | 0 (0 to 0) | 0 (0 to 0) | 0 (0 to 0) | 0 (0 to 0) | 0 (0 to 0) | 0 (0 to 0) | 0 (0 to 0) | 0 (0 to 0) | 0 (0 to 0) |
| Median HD (Q1 to Q3) 2nts 3’ end | 0 (0 to 0) | NR0 (0 to 0) | 0 (0 to 0) | 0 (0 to 0) | NR0 (0 to 0) | 0 (0 to 0) | 0 (0 to 0) | NR0 (0 to 0) | 0 (0 to 0) |
| Percent Qualified Definition #1 | 100% | 100% | 99.74% | 99.89% | 99.74% | 99.93% | 98.84% | 98.64% | 99.32% |
| Percent Qualified Definition #2 | 100% | NR100% | 99.74% | 99.78% | NR99.74% | 99.93% | 98.79% | NR98.64% | 99.20% |
| Percent Qualified Definition #3 | 95.89% | 100% | 89.06% | 87.18% | 91.75% | 95.63% | 88.83% | 93.16% | 84.39% |
| NR - Value not relevant for analysis | | | | | | | | | |
IPDA-BC Subtype-Matched Primers, psi
(b)
| Subtype (Country Code) | C (IN) | | | C (ZA) | | | B (US) | | |
| --- | --- | --- | --- | --- | --- | --- | --- | --- | --- |
| Sequence Name | forward | probe | reverse-C | forward | probe | reverse-C | forward | probe | reverse-B |
| Target Sequence 5’-3’ | TCTCGACGCAGGACTCG | GAGGCTAGAAGGAGAGAG | GGTGCGAGAGCGTCAATA | TCTCGACGCAGGACTCG | GAGGCTAGAAGGAGAGAG | GGTGCGAGAGCGTCAATA | TCTCGACGCAGGACTCG | GAGGCTAGAAGGAGAGAG | GGTGCGAGAGCGTCAGTA |
| Target Coordinates (HXB2) | 684-700 | 772-789 | 793-810 | 684-700 | 772-789 | 793-810 | 684-700 | 772-789 | 793-810 |
| Sample Size (Genomes) | 73 | 76 | 384 | 928 | 1176 | 5694 | 16801 | 18134 | 26028 |
| Median HD (Q1 to Q3) Full Length | 0 (0 to 0) | 0 (0 to 0) | 0 (0 to 0) | 0 (0 to 0) | 0 (0 to 0) | 0 (0 to 1) | 0 (0 to 0) | 0 (0 to 0) | 0 (0 to 1) |
| Median HD (Q1 to Q3) 5' Half | 0 (0 to 0) | 0 (0 to 0) | 0 (0 to 0) | 0 (0 to 0) | 0 (0 to 0) | 0 (0 to 1) | 0 (0 to 0) | 0 (0 to 0) | 0 (0 to 1) |
| Median HD (Q1 to Q3) 3' Half | 0 (0 to 0) | 0 (0 to 0) | 0 (0 to 0) | 0 (0 to 0) | 0 (0 to 0) | 0 (0 to 0) | 0 (0 to 0) | 0 (0 to 0) | 0 (0 to 0) |
| Median HD (Q1 to Q3) 2nts 3’ end | 0 (0 to 0) | NR0 (0 to 0) | 0 (0 to 0) | 0 (0 to 0) | 0 (0 to 0) | 0 (0 to 0) | 0 (0 to 0) | NR0 (0 to 0) | 0 (0 to 0) |
| Percent Qualified Definition #1 | 100% | 100% | 99.74% | 99.89% | 99.74% | 99.93% | 98.84% | 98.64% | 99.32% |
| Percent Qualified Definition #2 | 100% | NR100% | 99.74% | 99.78% | NR99.74% | 99.93% | 98.79% | NR98.64% | 99.21% |
| Percent Qualified Definition #3 | 95.89% | 100% | 77.60% | 87.18% | 91.75% | 61.36% | 88.83% | 93.16% | 68.86% |
| NR - Value not relevant for analysis | | | | | | | | | |
IPDA-BC Subtype-Mixed Primers, env
(c)
| Subtype (Country Code) | C (IN) | | | | C (ZA) | | | | B (US) | | | |
| --- | --- | --- | --- | --- | --- | --- | --- | --- | --- | --- | --- | --- |
| Sequence Name | forward-B&C | \*hypermutated probe | intact probe | \*reverse | forward-B&C | \*hypermutated probe | intact probe | \*reverse | forward-B&C | \*hypermutated probe | intact probe | \*reverse |
| Target Sequence 5’-3’ | AGTGGTGSAGAGAGAAAAAAGAGC | CCTTAGGTTCTTAGGAGC | CCTTGGGTTCTTGGGAGC | GCTGACGGTACAGGCCAGAC | AGTGGTGSAGAGAGAAAAAAGAGC | CCTTAGGTTCTTAGGAGC | CCTTGGGTTCTTGGGAGC | GCTGACGGTACAGGCCAGAC | AGTGGTGSAGAGAGAAAAAAGAGC | CCTTAGGTTCTTAGGAGC | CCTTGGGTTCTTGGGAGC | GCTGACGGTACAGGCCAGAC |
| Target Coordinates (HXB2) | 7736-7759 | 7781-7798 | 7781-7798 | 7832-7851 | 7736-7759 | 7781-7798 | 7781-7798 | 7832-7851 | 7736-7759 | 7781-7798 | 7781-7798 | 7832-7851 |
| Sample Size (Genomes) | 1176 | 1229 | | 1246 | 10049 | 10069 | | 10031 | 58806 | 56654 | | 55878 |
| Median HD (Q1 to Q3) Full Length | 0 (0 to 1) | 2 (2 to 3) | 0 (0 to 1) | 0 (0 to 0) | 0 (0 to 1) | 2 (2 to 2) | 0 (0 to 1) | 0 (0 to 1) | 0 (0 to 1) | 2 (2 to 2) | 0 (0 to 1) | 0 (0 to 1) |
| Median HD (Q1 to Q3) 5' Half | 0 (0 to 1) | 1 (1 to 1) | 0 (0 to 0) | 0 (0 to 0) | 0 (0 to 0) | 1 (1 to 1) | 0 (0 to 0) | 0 (0 to 1) | 0 (0 to 0) | 1 (1 to 1) | 0 (0 to 0) | 0 (0 to 1) |
| Median HD (Q1 to Q3) 3' Half | 0 (0 to 1) | 1 (1 to 1) | 0 (0 to 0) | 0 (0 to 0) | 0 (0 to 1) | 1 (1 to 1) | 0 (0 to 0) | 0 (0 to 0) | 0 (0 to 0) | 1 (1 to 1) | 0 (0 to 0) | 0 (0 to 0) |
| Median HD (Q1 to Q3) 2nts 3’ end | 0 (0 to 0) | NR0 (0 to 0) | NR0 (0 to 0) | 0 (0 to 0) | 0 (0 to 0) | NR0 (0 to 0) | NR0 (0 to 0) | 0 (0 to 0) | 0 (0 to 0) | NR0 (0 to 0) | NR0 (0 to 0) | 0 (0 to 0) |
| Percent Qualified Definition #1 | 89.71% | g90.64% | | 99.68% | 95.60% | h91.91% | | 99.66% | 97.25% | i93.43% | | 99.97% |
| Percent Qualified Definition #2 | 89.03% | NR90.64% | | 99.36% | 94.78% | NR91.91% | | 98.95% | 95.28% | NR93.43% | | 96.81% |
| Percent Qualified Definition #3 | 52.98% | 0% | 63.14% | 78.89% | 63.34% | 0.03% | 68.05% | 67.71% | 64.18% | 0.21% | 72.99% | 69.67% |
| NR – Value not relevant for analysis – Analysis identical to IPDA Original | | | | | | | | | | | | |
(d)
IPDA-BC Subtype-Matched Primers, env
| Subtype (Country Code) | C (IN) | | | | C (ZA) | | | | B (US) | | | |
| --- | --- | --- | --- | --- | --- | --- | --- | --- | --- | --- | --- | --- |
| Sequence Name | forward-C | \*hypermutated probe | intact probe | \*reverse | forward-C | \*hypermutated probe | intact probe | \*reverse | \*forward-B | \*hypermutated probe | intact probe | \*reverse |
| Target Sequence 5’-3’ | AGTGGTGGAGAGAGAAAAAAGAGC | CCTTAGGTTCTTAGGAGC | CCTTGGGTTCTTGGGAGC | GCTGACGGTACAGGCCAGAC | AGTGGTGGAGAGAGAAAAAAGAGC | CCTTAGGTTCTTAGGAGC | CCTTGGGTTCTTGGGAGC | GCTGACGGTACAGGCCAGAC | AGTGGTGCAGAGAGAAAAAAGAGC | CCTTAGGTTCTTAGGAGC | CCTTGGGTTCTTGGGAGC | GCTGACGGTACAGGCCAGAC |
| Target Coordinates (HXB2) | 7736-7759 | 7781-7798 | 7781-7798 | 7832-7851 | 7736-7759 | 7781-7798 | 7781-7798 | 7832-7851 | 7736-7759 | 7781-7798 | 7781-7798 | 7832-7851 |
| Sample Size (Genomes) | 1176 | 1229 | | 1246 | 10049 | 10069 | | 10031 | 58806 | 56654 | | 55878 |
| Median HD (Q1 to Q3) Full Length | 0 (0 to 1) | 2 (2 to 3) | 0 (0 to 1) | 0 (0 to 0) | 0 (0 to 1) | 2 (2 to 2) | 0 (0 to 1) | 0 (0 to 1) | 0 (0 to 1) | 2 (2 to 2) | 0 (0 to 1) | 0 (0 to 1) |
| Median HD (Q1 to Q3) 5' Half | 0 (0 to 1) | 1 (1 to 1) | 0 (0 to 0) | 0 (0 to 0) | 0 (0 to 0) | 1 (1 to 1) | 0 (0 to 0) | 0 (0 to 1) | 0 (0 to 0) | 1 (1 to 1) | 0 (0 to 0) | 0 (0 to 1) |
| Median HD (Q1 to Q3) 3' Half | 0 (0 to 1) | 1 (1 to 1) | 0 (0 to 0) | 0 (0 to 0) | 0 (0 to 1) | 1 (1 to 1) | 0 (0 to 0) | 0 (0 to 0) | 0 (0 to 0) | 1 (1 to 1) | 0 (0 to 0) | 0 (0 to 0) |
| Median HD (Q1 to Q3) 2nts 3’ end | 0 (0 to 0) | NR0 (0 to 0) | NR0 (0 to 0) | 0 (0 to 0) | 0 (0 to 0) | 0 (0 to 0) | 0 (0 to 0) | 0 (0 to 0) | 0 (0 to 0) | NR0 (0 to 0) | NR0 (0 to 0) | 0 (0 to 0) |
| Percent Qualified Definition #1 | 89.71% | j90.64% | | 99.68% | 95.60% | k91.91% | | 99.66% | 97.25% | l93.43% | | 99.97% |
| Percent Qualified Definition #2 | 89.03% | NR90.64% | | 99.36% | 94.78% | NR91.91% | | 98.95% | 95.28% | NR93.43% | | 96.81% |
| Percent Qualified Definition #3 | 52.13% | 0% | 63.14% | 78.89% | 63.07% | 0.03% | 68.05% | 67.71% | 64.17% | 0.21% | 72.99% | 69.67% |
| NR – Value not relevant for analysis – Analysis identical to IPDA Original | | | | | | | | | | | | |

## Slide 20
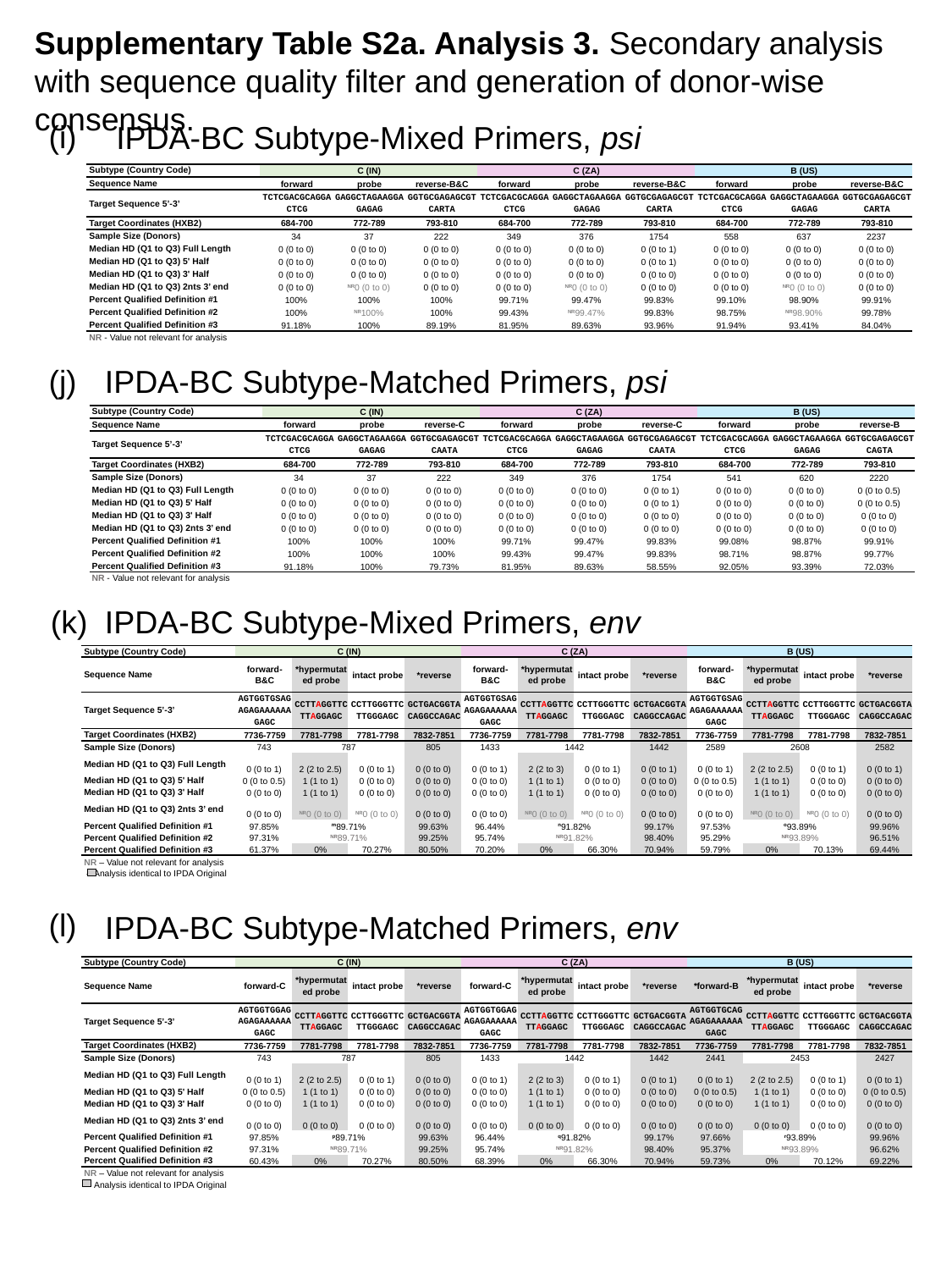

Supplementary Table S2a. Analysis 3. Secondary analysis with sequence quality filter and generation of donor-wise consensus.
(i)
IPDA-BC Subtype-Mixed Primers, psi
| Subtype (Country Code) | C (IN) | | | C (ZA) | | | B (US) | | |
| --- | --- | --- | --- | --- | --- | --- | --- | --- | --- |
| Sequence Name | forward | probe | reverse-B&C | forward | probe | reverse-B&C | forward | probe | reverse-B&C |
| Target Sequence 5’-3’ | TCTCGACGCAGGACTCG | GAGGCTAGAAGGAGAGAG | GGTGCGAGAGCGTCARTA | TCTCGACGCAGGACTCG | GAGGCTAGAAGGAGAGAG | GGTGCGAGAGCGTCARTA | TCTCGACGCAGGACTCG | GAGGCTAGAAGGAGAGAG | GGTGCGAGAGCGTCARTA |
| Target Coordinates (HXB2) | 684-700 | 772-789 | 793-810 | 684-700 | 772-789 | 793-810 | 684-700 | 772-789 | 793-810 |
| Sample Size (Donors) | 34 | 37 | 222 | 349 | 376 | 1754 | 558 | 637 | 2237 |
| Median HD (Q1 to Q3) Full Length | 0 (0 to 0) | 0 (0 to 0) | 0 (0 to 0) | 0 (0 to 0) | 0 (0 to 0) | 0 (0 to 1) | 0 (0 to 0) | 0 (0 to 0) | 0 (0 to 0) |
| Median HD (Q1 to Q3) 5' Half | 0 (0 to 0) | 0 (0 to 0) | 0 (0 to 0) | 0 (0 to 0) | 0 (0 to 0) | 0 (0 to 1) | 0 (0 to 0) | 0 (0 to 0) | 0 (0 to 0) |
| Median HD (Q1 to Q3) 3' Half | 0 (0 to 0) | 0 (0 to 0) | 0 (0 to 0) | 0 (0 to 0) | 0 (0 to 0) | 0 (0 to 0) | 0 (0 to 0) | 0 (0 to 0) | 0 (0 to 0) |
| Median HD (Q1 to Q3) 2nts 3’ end | 0 (0 to 0) | NR0 (0 to 0) | 0 (0 to 0) | 0 (0 to 0) | NR0 (0 to 0) | 0 (0 to 0) | 0 (0 to 0) | NR0 (0 to 0) | 0 (0 to 0) |
| Percent Qualified Definition #1 | 100% | 100% | 100% | 99.71% | 99.47% | 99.83% | 99.10% | 98.90% | 99.91% |
| Percent Qualified Definition #2 | 100% | NR100% | 100% | 99.43% | NR99.47% | 99.83% | 98.75% | NR98.90% | 99.78% |
| Percent Qualified Definition #3 | 91.18% | 100% | 89.19% | 81.95% | 89.63% | 93.96% | 91.94% | 93.41% | 84.04% |
| NR - Value not relevant for analysis | | | | | | | | | |
IPDA-BC Subtype-Matched Primers, psi
(j)
| Subtype (Country Code) | C (IN) | | | C (ZA) | | | B (US) | | |
| --- | --- | --- | --- | --- | --- | --- | --- | --- | --- |
| Sequence Name | forward | probe | reverse-C | forward | probe | reverse-C | forward | probe | reverse-B |
| Target Sequence 5’-3’ | TCTCGACGCAGGACTCG | GAGGCTAGAAGGAGAGAG | GGTGCGAGAGCGTCAATA | TCTCGACGCAGGACTCG | GAGGCTAGAAGGAGAGAG | GGTGCGAGAGCGTCAATA | TCTCGACGCAGGACTCG | GAGGCTAGAAGGAGAGAG | GGTGCGAGAGCGTCAGTA |
| Target Coordinates (HXB2) | 684-700 | 772-789 | 793-810 | 684-700 | 772-789 | 793-810 | 684-700 | 772-789 | 793-810 |
| Sample Size (Donors) | 34 | 37 | 222 | 349 | 376 | 1754 | 541 | 620 | 2220 |
| Median HD (Q1 to Q3) Full Length | 0 (0 to 0) | 0 (0 to 0) | 0 (0 to 0) | 0 (0 to 0) | 0 (0 to 0) | 0 (0 to 1) | 0 (0 to 0) | 0 (0 to 0) | 0 (0 to 0.5) |
| Median HD (Q1 to Q3) 5' Half | 0 (0 to 0) | 0 (0 to 0) | 0 (0 to 0) | 0 (0 to 0) | 0 (0 to 0) | 0 (0 to 1) | 0 (0 to 0) | 0 (0 to 0) | 0 (0 to 0.5) |
| Median HD (Q1 to Q3) 3' Half | 0 (0 to 0) | 0 (0 to 0) | 0 (0 to 0) | 0 (0 to 0) | 0 (0 to 0) | 0 (0 to 0) | 0 (0 to 0) | 0 (0 to 0) | 0 (0 to 0) |
| Median HD (Q1 to Q3) 2nts 3’ end | 0 (0 to 0) | 0 (0 to 0) | 0 (0 to 0) | 0 (0 to 0) | 0 (0 to 0) | 0 (0 to 0) | 0 (0 to 0) | 0 (0 to 0) | 0 (0 to 0) |
| Percent Qualified Definition #1 | 100% | 100% | 100% | 99.71% | 99.47% | 99.83% | 99.08% | 98.87% | 99.91% |
| Percent Qualified Definition #2 | 100% | 100% | 100% | 99.43% | 99.47% | 99.83% | 98.71% | 98.87% | 99.77% |
| Percent Qualified Definition #3 | 91.18% | 100% | 79.73% | 81.95% | 89.63% | 58.55% | 92.05% | 93.39% | 72.03% |
| NR - Value not relevant for analysis | | | | | | | | | |
(k)
IPDA-BC Subtype-Mixed Primers, env
| Subtype (Country Code) | C (IN) | | | | C (ZA) | | | | B (US) | | | |
| --- | --- | --- | --- | --- | --- | --- | --- | --- | --- | --- | --- | --- |
| Sequence Name | forward-B&C | \*hypermutated probe | intact probe | \*reverse | forward-B&C | \*hypermutated probe | intact probe | \*reverse | forward-B&C | \*hypermutated probe | intact probe | \*reverse |
| Target Sequence 5’-3’ | AGTGGTGSAGAGAGAAAAAAGAGC | CCTTAGGTTCTTAGGAGC | CCTTGGGTTCTTGGGAGC | GCTGACGGTACAGGCCAGAC | AGTGGTGSAGAGAGAAAAAAGAGC | CCTTAGGTTCTTAGGAGC | CCTTGGGTTCTTGGGAGC | GCTGACGGTACAGGCCAGAC | AGTGGTGSAGAGAGAAAAAAGAGC | CCTTAGGTTCTTAGGAGC | CCTTGGGTTCTTGGGAGC | GCTGACGGTACAGGCCAGAC |
| Target Coordinates (HXB2) | 7736-7759 | 7781-7798 | 7781-7798 | 7832-7851 | 7736-7759 | 7781-7798 | 7781-7798 | 7832-7851 | 7736-7759 | 7781-7798 | 7781-7798 | 7832-7851 |
| Sample Size (Donors) | 743 | 787 | | 805 | 1433 | 1442 | | 1442 | 2589 | 2608 | | 2582 |
| Median HD (Q1 to Q3) Full Length | 0 (0 to 1) | 2 (2 to 2.5) | 0 (0 to 1) | 0 (0 to 0) | 0 (0 to 1) | 2 (2 to 3) | 0 (0 to 1) | 0 (0 to 1) | 0 (0 to 1) | 2 (2 to 2.5) | 0 (0 to 1) | 0 (0 to 1) |
| Median HD (Q1 to Q3) 5' Half | 0 (0 to 0.5) | 1 (1 to 1) | 0 (0 to 0) | 0 (0 to 0) | 0 (0 to 0) | 1 (1 to 1) | 0 (0 to 0) | 0 (0 to 0) | 0 (0 to 0.5) | 1 (1 to 1) | 0 (0 to 0) | 0 (0 to 0) |
| Median HD (Q1 to Q3) 3' Half | 0 (0 to 0) | 1 (1 to 1) | 0 (0 to 0) | 0 (0 to 0) | 0 (0 to 0) | 1 (1 to 1) | 0 (0 to 0) | 0 (0 to 0) | 0 (0 to 0) | 1 (1 to 1) | 0 (0 to 0) | 0 (0 to 0) |
| Median HD (Q1 to Q3) 2nts 3’ end | 0 (0 to 0) | NR0 (0 to 0) | NR0 (0 to 0) | 0 (0 to 0) | 0 (0 to 0) | NR0 (0 to 0) | NR0 (0 to 0) | 0 (0 to 0) | 0 (0 to 0) | NR0 (0 to 0) | NR0 (0 to 0) | 0 (0 to 0) |
| Percent Qualified Definition #1 | 97.85% | m89.71% | | 99.63% | 96.44% | n91.82% | | 99.17% | 97.53% | o93.89% | | 99.96% |
| Percent Qualified Definition #2 | 97.31% | NR89.71% | | 99.25% | 95.74% | NR91.82% | | 98.40% | 95.29% | NR93.89% | | 96.51% |
| Percent Qualified Definition #3 | 61.37% | 0% | 70.27% | 80.50% | 70.20% | 0% | 66.30% | 70.94% | 59.79% | 0% | 70.13% | 69.44% |
| NR – Value not relevant for analysis – Analysis identical to IPDA Original | | | | | | | | | | | | |
(l)
IPDA-BC Subtype-Matched Primers, env
| Subtype (Country Code) | C (IN) | | | | C (ZA) | | | | B (US) | | | |
| --- | --- | --- | --- | --- | --- | --- | --- | --- | --- | --- | --- | --- |
| Sequence Name | forward-C | \*hypermutated probe | intact probe | \*reverse | forward-C | \*hypermutated probe | intact probe | \*reverse | \*forward-B | \*hypermutated probe | intact probe | \*reverse |
| Target Sequence 5’-3’ | AGTGGTGGAGAGAGAAAAAAGAGC | CCTTAGGTTCTTAGGAGC | CCTTGGGTTCTTGGGAGC | GCTGACGGTACAGGCCAGAC | AGTGGTGGAGAGAGAAAAAAGAGC | CCTTAGGTTCTTAGGAGC | CCTTGGGTTCTTGGGAGC | GCTGACGGTACAGGCCAGAC | AGTGGTGCAGAGAGAAAAAAGAGC | CCTTAGGTTCTTAGGAGC | CCTTGGGTTCTTGGGAGC | GCTGACGGTACAGGCCAGAC |
| Target Coordinates (HXB2) | 7736-7759 | 7781-7798 | 7781-7798 | 7832-7851 | 7736-7759 | 7781-7798 | 7781-7798 | 7832-7851 | 7736-7759 | 7781-7798 | 7781-7798 | 7832-7851 |
| Sample Size (Donors) | 743 | 787 | | 805 | 1433 | 1442 | | 1442 | 2441 | 2453 | | 2427 |
| Median HD (Q1 to Q3) Full Length | 0 (0 to 1) | 2 (2 to 2.5) | 0 (0 to 1) | 0 (0 to 0) | 0 (0 to 1) | 2 (2 to 3) | 0 (0 to 1) | 0 (0 to 1) | 0 (0 to 1) | 2 (2 to 2.5) | 0 (0 to 1) | 0 (0 to 1) |
| Median HD (Q1 to Q3) 5' Half | 0 (0 to 0.5) | 1 (1 to 1) | 0 (0 to 0) | 0 (0 to 0) | 0 (0 to 0) | 1 (1 to 1) | 0 (0 to 0) | 0 (0 to 0) | 0 (0 to 0.5) | 1 (1 to 1) | 0 (0 to 0) | 0 (0 to 0.5) |
| Median HD (Q1 to Q3) 3' Half | 0 (0 to 0) | 1 (1 to 1) | 0 (0 to 0) | 0 (0 to 0) | 0 (0 to 0) | 1 (1 to 1) | 0 (0 to 0) | 0 (0 to 0) | 0 (0 to 0) | 1 (1 to 1) | 0 (0 to 0) | 0 (0 to 0) |
| Median HD (Q1 to Q3) 2nts 3’ end | 0 (0 to 0) | 0 (0 to 0) | 0 (0 to 0) | 0 (0 to 0) | 0 (0 to 0) | 0 (0 to 0) | 0 (0 to 0) | 0 (0 to 0) | 0 (0 to 0) | 0 (0 to 0) | 0 (0 to 0) | 0 (0 to 0) |
| Percent Qualified Definition #1 | 97.85% | p89.71% | | 99.63% | 96.44% | q91.82% | | 99.17% | 97.66% | r93.89% | | 99.96% |
| Percent Qualified Definition #2 | 97.31% | NR89.71% | | 99.25% | 95.74% | NR91.82% | | 98.40% | 95.37% | NR93.89% | | 96.62% |
| Percent Qualified Definition #3 | 60.43% | 0% | 70.27% | 80.50% | 68.39% | 0% | 66.30% | 70.94% | 59.73% | 0% | 70.12% | 69.22% |
| NR – Value not relevant for analysis – Analysis identical to IPDA Original | | | | | | | | | | | | |

## Slide 21
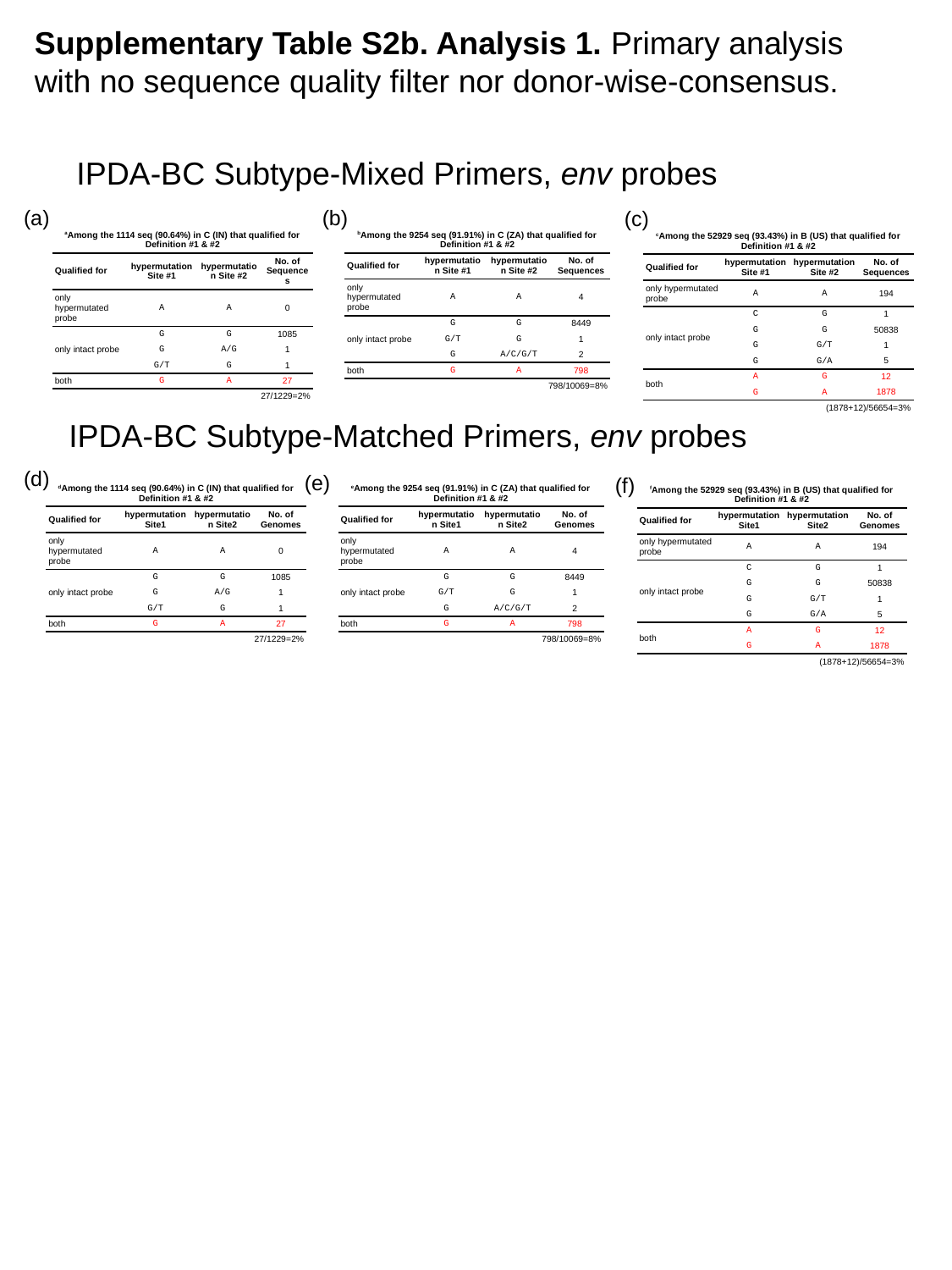

Supplementary Table S2b. Analysis 1. Primary analysis with no sequence quality filter nor donor-wise-consensus.
IPDA-BC Subtype-Mixed Primers, env probes
(a)
(b)
(c)
| aAmong the 1114 seq (90.64%) in C (IN) that qualified for Definition #1 & #2 | | | |
| --- | --- | --- | --- |
| Qualified for | hypermutation Site #1 | hypermutation Site #2 | No. of Sequences |
| only hypermutated probe | A | A | 0 |
| only intact probe | G | G | 1085 |
| | G | A/G | 1 |
| | G/T | G | 1 |
| both | G | A | 27 |
| 27/1229=2% | | | |
| bAmong the 9254 seq (91.91%) in C (ZA) that qualified for Definition #1 & #2 | | | |
| --- | --- | --- | --- |
| Qualified for | hypermutation Site #1 | hypermutation Site #2 | No. of Sequences |
| only hypermutated probe | A | A | 4 |
| only intact probe | G | G | 8449 |
| | G/T | G | 1 |
| | G | A/C/G/T | 2 |
| both | G | A | 798 |
| 798/10069=8% | | | |
| cAmong the 52929 seq (93.43%) in B (US) that qualified for Definition #1 & #2 | | | |
| --- | --- | --- | --- |
| Qualified for | hypermutation Site #1 | hypermutation Site #2 | No. of Sequences |
| only hypermutated probe | A | A | 194 |
| only intact probe | C | G | 1 |
| | G | G | 50838 |
| | G | G/T | 1 |
| | G | G/A | 5 |
| both | A | G | 12 |
| | G | A | 1878 |
| (1878+12)/56654=3% | | | |
IPDA-BC Subtype-Matched Primers, env probes
(d)
(e)
(f)
| dAmong the 1114 seq (90.64%) in C (IN) that qualified for Definition #1 & #2 | | | |
| --- | --- | --- | --- |
| Qualified for | hypermutation Site1 | hypermutation Site2 | No. of Genomes |
| only hypermutated probe | A | A | 0 |
| only intact probe | G | G | 1085 |
| | G | A/G | 1 |
| | G/T | G | 1 |
| both | G | A | 27 |
| 27/1229=2% | | | |
| eAmong the 9254 seq (91.91%) in C (ZA) that qualified for Definition #1 & #2 | | | |
| --- | --- | --- | --- |
| Qualified for | hypermutation Site1 | hypermutation Site2 | No. of Genomes |
| only hypermutated probe | A | A | 4 |
| only intact probe | G | G | 8449 |
| | G/T | G | 1 |
| | G | A/C/G/T | 2 |
| both | G | A | 798 |
| 798/10069=8% | | | |
| fAmong the 52929 seq (93.43%) in B (US) that qualified for Definition #1 & #2 | | | |
| --- | --- | --- | --- |
| Qualified for | hypermutation Site1 | hypermutation Site2 | No. of Genomes |
| only hypermutated probe | A | A | 194 |
| only intact probe | C | G | 1 |
| | G | G | 50838 |
| | G | G/T | 1 |
| | G | G/A | 5 |
| both | A | G | 12 |
| | G | A | 1878 |
| (1878+12)/56654=3% | | | |

## Slide 22
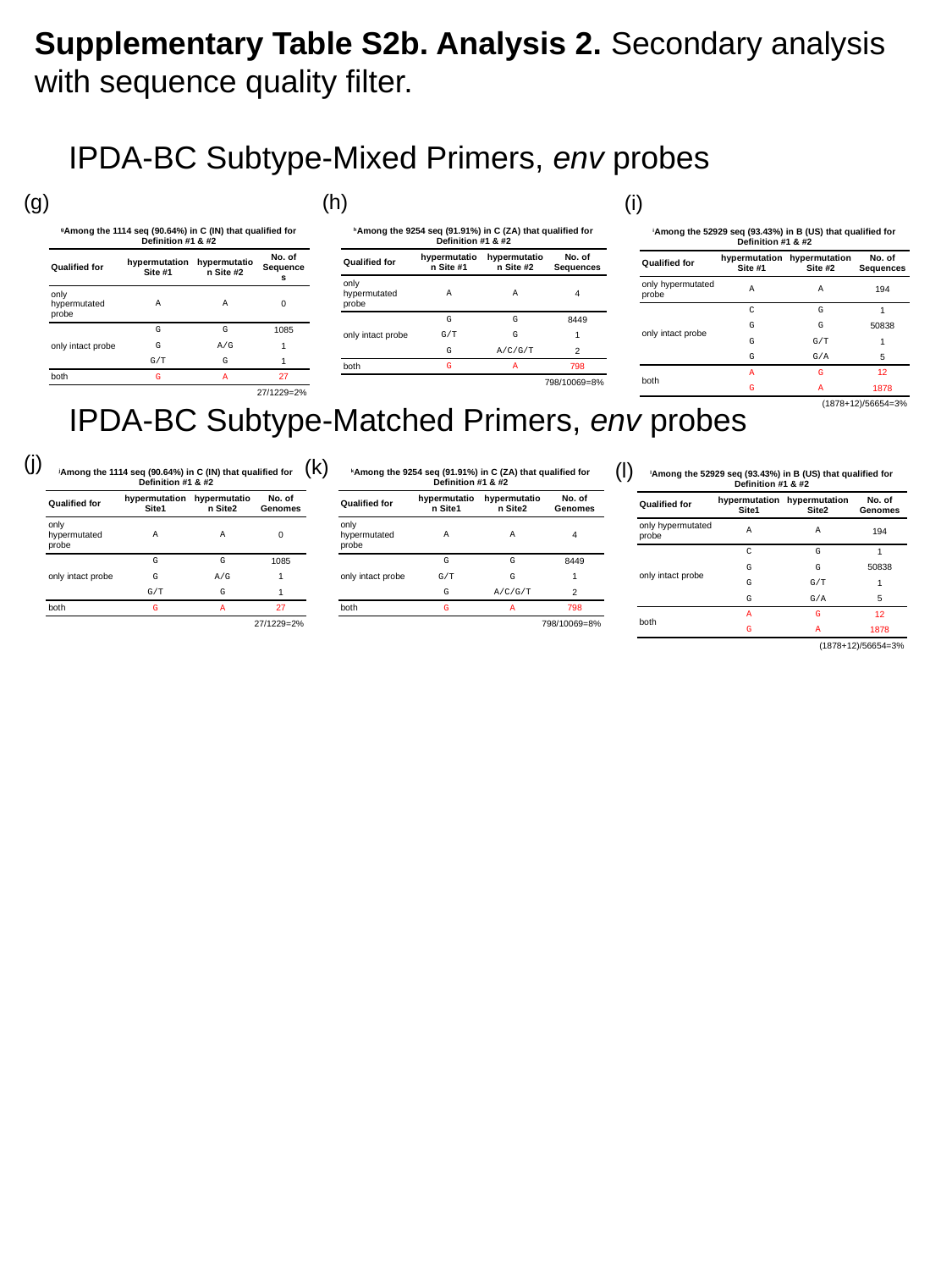

Supplementary Table S2b. Analysis 2. Secondary analysis with sequence quality filter.
IPDA-BC Subtype-Mixed Primers, env probes
(g)
(h)
(i)
| gAmong the 1114 seq (90.64%) in C (IN) that qualified for Definition #1 & #2 | | | |
| --- | --- | --- | --- |
| Qualified for | hypermutation Site #1 | hypermutation Site #2 | No. of Sequences |
| only hypermutated probe | A | A | 0 |
| only intact probe | G | G | 1085 |
| | G | A/G | 1 |
| | G/T | G | 1 |
| both | G | A | 27 |
| 27/1229=2% | | | |
| hAmong the 9254 seq (91.91%) in C (ZA) that qualified for Definition #1 & #2 | | | |
| --- | --- | --- | --- |
| Qualified for | hypermutation Site #1 | hypermutation Site #2 | No. of Sequences |
| only hypermutated probe | A | A | 4 |
| only intact probe | G | G | 8449 |
| | G/T | G | 1 |
| | G | A/C/G/T | 2 |
| both | G | A | 798 |
| 798/10069=8% | | | |
| iAmong the 52929 seq (93.43%) in B (US) that qualified for Definition #1 & #2 | | | |
| --- | --- | --- | --- |
| Qualified for | hypermutation Site #1 | hypermutation Site #2 | No. of Sequences |
| only hypermutated probe | A | A | 194 |
| only intact probe | C | G | 1 |
| | G | G | 50838 |
| | G | G/T | 1 |
| | G | G/A | 5 |
| both | A | G | 12 |
| | G | A | 1878 |
| (1878+12)/56654=3% | | | |
IPDA-BC Subtype-Matched Primers, env probes
(j)
(k)
(l)
| jAmong the 1114 seq (90.64%) in C (IN) that qualified for Definition #1 & #2 | | | |
| --- | --- | --- | --- |
| Qualified for | hypermutation Site1 | hypermutation Site2 | No. of Genomes |
| only hypermutated probe | A | A | 0 |
| only intact probe | G | G | 1085 |
| | G | A/G | 1 |
| | G/T | G | 1 |
| both | G | A | 27 |
| 27/1229=2% | | | |
| kAmong the 9254 seq (91.91%) in C (ZA) that qualified for Definition #1 & #2 | | | |
| --- | --- | --- | --- |
| Qualified for | hypermutation Site1 | hypermutation Site2 | No. of Genomes |
| only hypermutated probe | A | A | 4 |
| only intact probe | G | G | 8449 |
| | G/T | G | 1 |
| | G | A/C/G/T | 2 |
| both | G | A | 798 |
| 798/10069=8% | | | |
| lAmong the 52929 seq (93.43%) in B (US) that qualified for Definition #1 & #2 | | | |
| --- | --- | --- | --- |
| Qualified for | hypermutation Site1 | hypermutation Site2 | No. of Genomes |
| only hypermutated probe | A | A | 194 |
| only intact probe | C | G | 1 |
| | G | G | 50838 |
| | G | G/T | 1 |
| | G | G/A | 5 |
| both | A | G | 12 |
| | G | A | 1878 |
| (1878+12)/56654=3% | | | |

## Slide 23
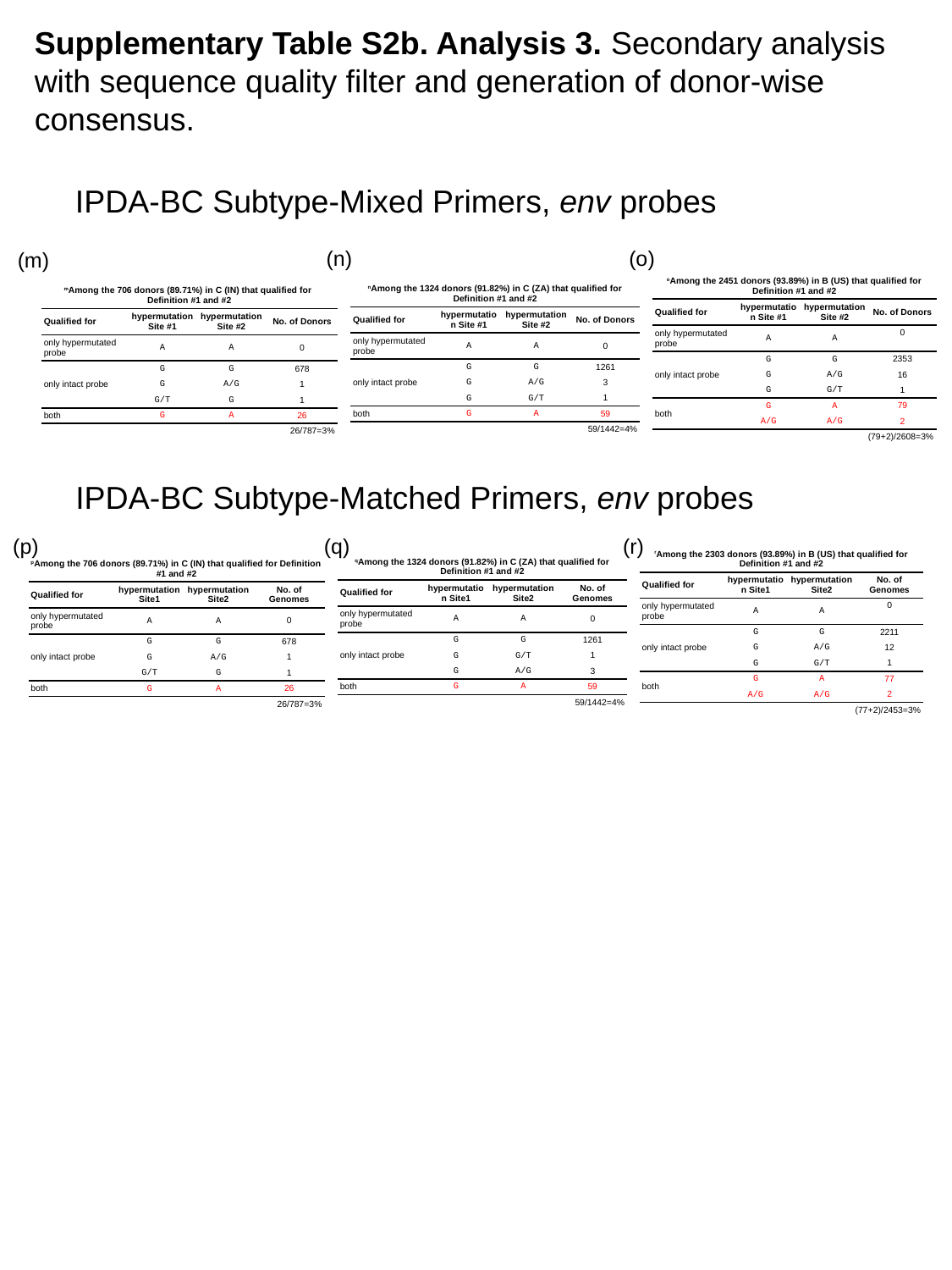

Supplementary Table S2b. Analysis 3. Secondary analysis with sequence quality filter and generation of donor-wise consensus.
IPDA-BC Subtype-Mixed Primers, env probes
(n)
(o)
(m)
| oAmong the 2451 donors (93.89%) in B (US) that qualified for Definition #1 and #2 | | | |
| --- | --- | --- | --- |
| Qualified for | hypermutation Site #1 | hypermutation Site #2 | No. of Donors |
| only hypermutated probe | A | A | 0 |
| only intact probe | G | G | 2353 |
| | G | A/G | 16 |
| | G | G/T | 1 |
| both | G | A | 79 |
| | A/G | A/G | 2 |
| (79+2)/2608=3% | | | |
| nAmong the 1324 donors (91.82%) in C (ZA) that qualified for Definition #1 and #2 | | | |
| --- | --- | --- | --- |
| Qualified for | hypermutation Site #1 | hypermutation Site #2 | No. of Donors |
| only hypermutated probe | A | A | 0 |
| only intact probe | G | G | 1261 |
| | G | A/G | 3 |
| | G | G/T | 1 |
| both | G | A | 59 |
| 59/1442=4% | | | |
| mAmong the 706 donors (89.71%) in C (IN) that qualified for Definition #1 and #2 | | | |
| --- | --- | --- | --- |
| Qualified for | hypermutation Site #1 | hypermutation Site #2 | No. of Donors |
| only hypermutated probe | A | A | 0 |
| only intact probe | G | G | 678 |
| | G | A/G | 1 |
| | G/T | G | 1 |
| both | G | A | 26 |
| 26/787=3% | | | |
IPDA-BC Subtype-Matched Primers, env probes
(p)
(q)
(r)
| rAmong the 2303 donors (93.89%) in B (US) that qualified for Definition #1 and #2 | | | |
| --- | --- | --- | --- |
| Qualified for | hypermutation Site1 | hypermutation Site2 | No. of Genomes |
| only hypermutated probe | A | A | 0 |
| only intact probe | G | G | 2211 |
| | G | A/G | 12 |
| | G | G/T | 1 |
| both | G | A | 77 |
| | A/G | A/G | 2 |
| (77+2)/2453=3% | | | |
| qAmong the 1324 donors (91.82%) in C (ZA) that qualified for Definition #1 and #2 | | | |
| --- | --- | --- | --- |
| Qualified for | hypermutation Site1 | hypermutation Site2 | No. of Genomes |
| only hypermutated probe | A | A | 0 |
| only intact probe | G | G | 1261 |
| | G | G/T | 1 |
| | G | A/G | 3 |
| both | G | A | 59 |
| 59/1442=4% | | | |
| pAmong the 706 donors (89.71%) in C (IN) that qualified for Definition #1 and #2 | | | |
| --- | --- | --- | --- |
| Qualified for | hypermutation Site1 | hypermutation Site2 | No. of Genomes |
| only hypermutated probe | A | A | 0 |
| only intact probe | G | G | 678 |
| | G | A/G | 1 |
| | G/T | G | 1 |
| both | G | A | 26 |
| 26/787=3% | | | |
